# Neural ensembles in the murine medial prefrontal cortex process distinct information during visual perceptual learning

**DOI:** 10.1186/s12915-023-01529-x

**Published:** 2023-02-24

**Authors:** Zhenni Wang, Shihao Lou, Xiao Ma, Hui Guo, Yan Liu, Wenjing Chen, Dating Lin, Yupeng Yang

**Affiliations:** 1 Division of Life Sciences and Medicine, Hefei National Research Center for Physical Sciences at the Microscale, University of Science and Technology of China, Hefei, 230026 China; 2 Intramural Research Program, National Institute On Drug Abuse, National Institutes of Health, Baltimore, MD 21224 USA

**Keywords:** Visual perceptual learning, Medial prefrontal cortex, Pyramidal neurons, Attention, Reward

## Abstract

**Background:**

Perceptual learning refers to an augmentation of an organism’s ability to respond to external stimuli, which has been described in most sensory modalities. Visual perceptual learning (VPL) is a manifestation of plasticity in visual information processing that occurs in the adult brain, and can be used to ameliorate the ability of patients with visual defects mainly based on an improvement of detection or discrimination of features in visual tasks. While some brain regions such as the primary visual cortex have been described to participate in VPL, the way more general high-level cognitive brain areas are involved in this process remains unclear. Here, we showed that the medial prefrontal cortex (mPFC) was essential for both the training and maintenance processes of VPL in mouse models.

**Results:**

We built a new VPL model in a custom-designed training chamber to enable the utilization of miniScopes when mice freely executed the VPL task. We found that pyramidal neurons in the mPFC participate in both the training process and maintenance of VPL. By recording the calcium activity of mPFC pyramidal neurons while mice freely executed the task, distinct ON and OFF neural ensembles tuned to different behaviors were identified, which might encode different cognitive information. Decoding analysis showed that mouse behaviors could be well predicted using the activity of each ON ensemble. Furthermore, VPL recruited more reward-related components in the mPFC.

**Conclusion:**

We revealed the neural mechanism underlying vision improvement following VPL and identify distinct ON and OFF neural ensembles in the mPFC that tuned to different information during visual perceptual training. These results uncover an important role of the mPFC in VPL, with more reward-related components being also involved, and pave the way for future clarification of the reward signal coding rules in VPL.

**Supplementary Information:**

The online version contains supplementary material available at 10.1186/s12915-023-01529-x.

## Background

Perceptual learning is a relatively long-lasting and experience-dependent enhancement of organisms’ ability to respond to the external environment [1, 2]. It has been documented in virtually all tasks and sensory modalities, such as vision, audition, touch, taste, and smell [3, 4]. Even the simplest task of detecting or discriminating a perceptual stimulus involves a network of processes or brain regions supporting sensory processing, decision, action selection, top-down task relevance, attention, and processing of rewards or feedback [3]. Visual perceptual learning (VPL), as one of the most commonly studied models of perceptual learning, has been proven to occur for different kinds of tasks at different levels. For example, VPL can improve detection or discrimination for single features (spatial frequency, orientation, phase, contrast, color, acuity, and hyperacuity), pattern discrimination of compound stimuli (textures, depth, and motion), and identification of objects and natural scenes (faces, shapes, and biological motion) [3, 5]. Training on visual tasks has been used to significantly strengthen the visual abilities of adult patients with amblyopia and other forms of abnormal vision [4, 6, 7]. However, the neural mechanisms underlying VPL are largely unknown.

Numerous physiological and psychophysical studies in mouse and nonhuman primate models suggest that VPL requires the involvement of the primary visual cortex (V1) [8–10], which is known to process more primitive visual signals more locally [11]. Evidence of functional and structural changes in V1 associated with task-relevant VPL has been reported in many studies, including ours [8, 12–14]. Meanwhile, human functional magnetic resonance imaging (fMRI) studies have shown that activations of some prefrontal regions are highly correlated with the magnitude of performance improvement over the course of perceptual training [15–17]. However, previous noninvasive studies cannot provide direct evidence about whether high-level cognitive areas are necessary for performance improvements in VPL. The medial prefrontal cortex (mPFC) plays an important role in many higher cognitive processes, such as attention [18], motivation [19], hidden state inference [20], reward evaluation [21], and decision making [22]. High-level cognitive components such as attention or reward have been proposed to be important driving factors of task-relevant VPL [23–26]. Numerous behavioral studies suggest that attention seems necessary for perceptual learning [10, 23, 27, 28], while a few studies show that perceptual learning can eliminate the limitations of attention [29, 30]. Similarly, rewards are thought to play an important role in activating perceptual learning. Task-irrelevant VPL occurs when the feature is paired with a reward and does not occur without a reward [24]. However, how different cognitive components in the mPFC are involved in VPL at the neuron level remains largely unknown.

The rodent mPFC is functionally homologous to the human and primate mPFC/DLPFC [31, 32]. Attention-, outcome-, motor-, and sensory-related activity have been recorded in rodent mPFC neurons during goal-directed behaviors [18, 33]. In the present study, we showed that the mouse mPFC was crucial to the process of VPL. To explore how cognitive functions such as attention and reward were involved in the VPL training by the miniature fluorescence microscope (miniScope), we designed a customized training chamber for a new mouse VPL model with a similar training procedure as that in the water task. Real-time neuronal calcium imaging further showed that pyramidal neurons in the mPFC formed distinct ensembles preferentially tuned to different task-related behavioral events. Furthermore, more reward-coding neurons in the mPFC were recruited after a period of VPL training. These results uncover an important role of the mPFC and reward-related neural ensemble in perceptual learning of spatial frequency.

## Results

### The mPFC contributes to both the training process and the maintenance of VPL

**Fig. 1:**
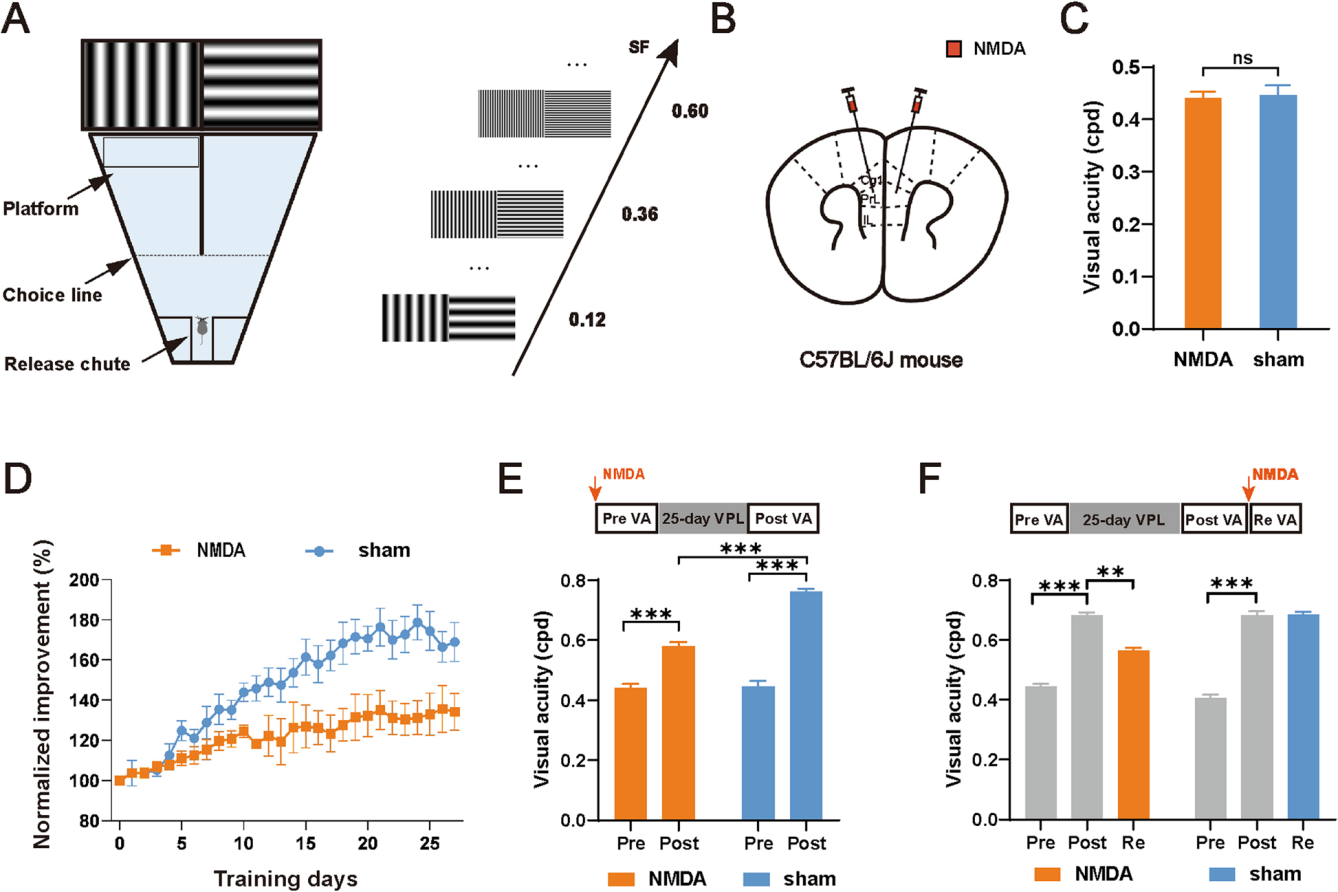
The mPFC is important to both the training process and the maintenance of VPL. **A** Diagram of the visual water task. Following release from the chute, the mice learned to swim toward the monitor displaying a vertical grating to find the submerged platform and escape from the water. **B** A schematic of NMDA injection in the mPFC. **C**. The pretraining VA for NMDA lesion and sham mice (NMDA lesion: 0.44 ± 0.03 cpd vs. sham: 0.45 ± 0.02 cpd, *p* > 0.05). NMDA, *n* = 11; sham, *n* = 5. **D** The average learning curve for mice across training days in the NMDA lesion and sham groups. The VA was normalized to the individual pretraining VA. NMDA, *n* = 11; sham, *n* = 5. **E** Top: schematic of the experimental procedure. Bottom: the pre- (Pre) and post-NSF training VA (Post) obtained from mice in the NMDA lesion group and sham group, respectively (NMDA: pre: 0.44 ± 0.03 cpd vs. post: 0.58 ± 0.04 cpd, *p* < 0.005, sham: pre: 0.45 ± 0.02 cpd vs. post: 0.76 ± 0.02 cpd, *p* < 0.005). NMDA, *n* = 11; sham, *n* = 5. **F** Top: schematic of the experimental procedure. Bottom: the pre- and post-NSF training VA as well as the retested VA after NMDA lesion (Re). NMDA, *n* = 5; sham, *n* = 5. Data are represented as the mean ± SEM. ∗ , *p* < 0.05; ∗  ∗ , *p* < 0.01; ∗  ∗  ∗ , *p* < 0.005; n.s., not significant (*p* > 0.05)

Prolonged training in a two-alternative forced choice visual water task near the individual threshold of spatial frequency (NSF) significantly improves the visual acuity (VA) of mice, which mimics the effect of VPL on amblyopic patients [12] (Fig. 1A). To investigate whether the mPFC participated in VPL, we bilaterally lesioned the mPFC with NMDA-induced excitotoxicity in adult male C57BL/6 J mice (Fig. 1B). Five days after NMDA injection, mPFC (mostly in Cg1, PrL and IL) neurons were ablated, while other brain areas remained intact (Additional file 1: Fig. S1A). Mice were trained to learn the visual water task and then underwent VA assessment. The lesioned group and the control group showed similar learning curves of the visual water task and pretraining VA (NMDA: 0.44 ± 0.01 cpd vs. sham: 0.45 ± 0.02 cpd, NMDA, *n* = 11; sham, *n* = 5, *p* > 0.05, *t* test, Fig. 1C and Additional file 1: Fig. S1B), suggesting that mPFC ablation does not impact the acquisition of the visual discrimination task. Then, both groups of mice received long-term training in the visual water task. The VA of the control mice gradually improved, and the average VA after training was 0.76 ± 0.01 cpd (*p* < 0.005, Fig. 1D, E). In contrast, mPFC-lesioned mice showed impaired improvement of VA during training, and the VA increased by 32% to 0.58 ± 0.01 cpd after training (*p* < 0.005, Fig. 1D, E). The posttraining VA of the lesioned group was significantly lower than that of the control group (*p* < 0.005, NMDA, *n* = 11; sham, *n* = 5, Fig. 1E).

Next, we examined whether the mPFC is also required for the maintenance of improved VA by VPL. We trained another group of naive mice and randomly divided them into two groups that were subjected to either bilateral lesions of the mPFC or sham operation. Five days after lesion induction, the VA of the trained mice was retested. We observed a significant decrease in the VA of the lesioned group and no significant change in the VA of the sham group (NMDA: Post: 0.68 ± 0.01 cpd vs. Re: 0.56 ± 0.01 cpd, *p* < 0.05, sham: Post: 0.68 ± 0.02 cpd vs. Re: 0.69 ± 0.01 cpd, NMDA, *n* = 5; sham, *n* = 5, *p* > 0.05, Fig. 1F). Collectively, these results indicate that the mPFC plays an important role in both the training process and the maintenance of VPL.

**Fig. 2:**
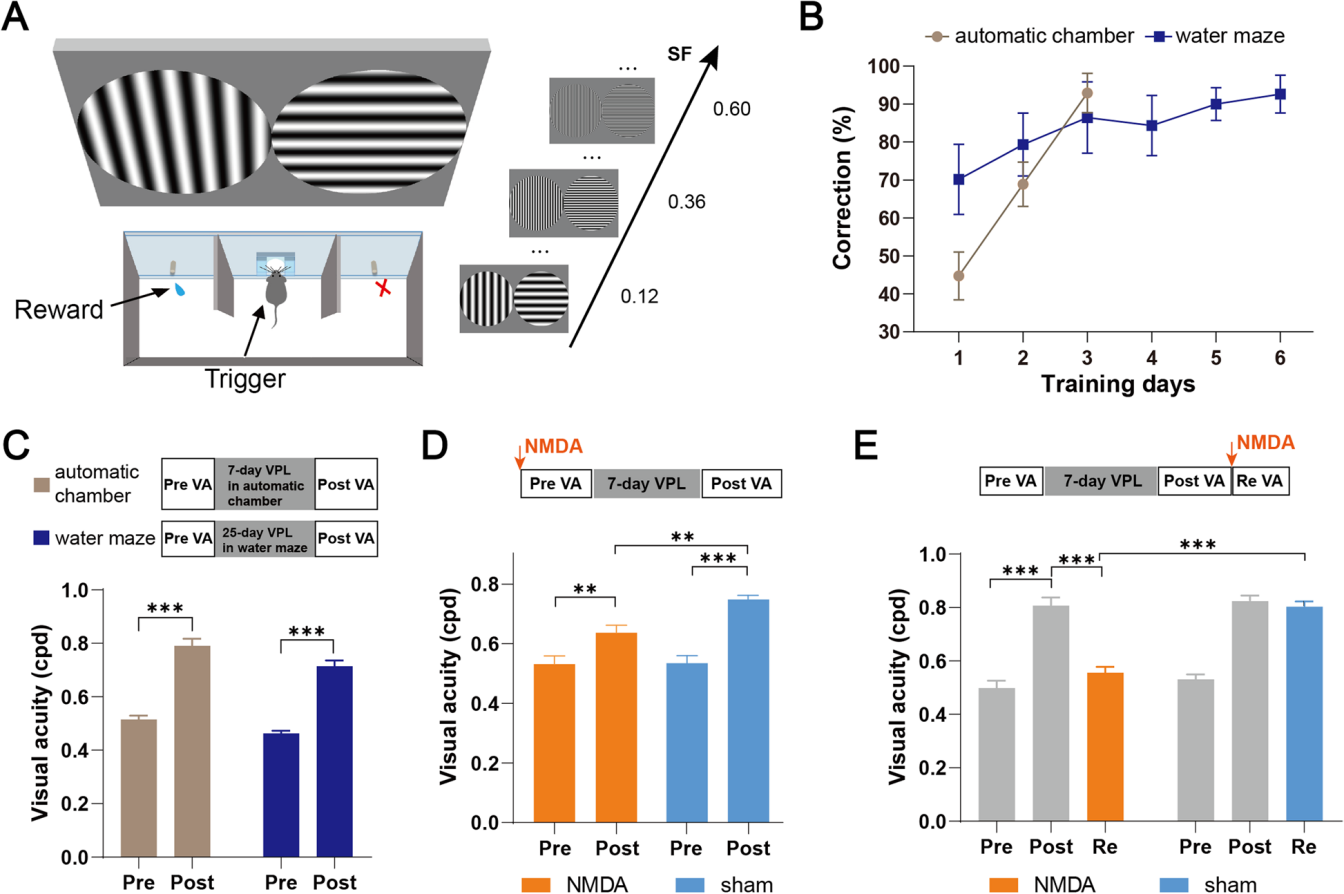
The mPFC participates in VPL training in the custom design training chamber. **A** Diagram of the custom-designed training chamber. **B** The correction rate across training days when mice acquired the visual discrimination task in the training chamber and water maze. Chamber, *n* = 12; water maze, *n* = 18. **C** Top: schematic of the experimental procedure in the automatic chamber. Middle: schematic of the experimental procedure in the water maze. Bottom: the pretraining VA (Pre) and posttraining VA (Post) for mice in the training chamber and water maze. Chamber, *n* = 12; water maze, *n* = 18. **D** Top: schematic of the experimental procedure. Bottom: the pretraining and posttraining VA obtained from mice in the NMDA lesion group and sham group, respectively. NMDA, *n* = 5; sham, *n* = 5. **E** Top: schematic of the experimental procedure. Bottom: the pretraining and posttraining VA as well as the retested VA (Re) after NMDA lesion. NMDA, *n* = 5; sham, *n* = 5. Data are represented as the mean ± SEM. ∗ , *p* < 0.05; ∗  ∗ , *p* < 0.01; ∗  ∗  ∗ , *p* < 0.005; n.s., not significant (*p* > 0.05)

To explore how mPFC neurons were involved in attention and reward-related behaviors in the VPL task, we designed a novel training chamber to build a new mouse VPL model based on goal-directed behavior in which mice need to trigger a trial actively and can receive a water reward in correct trials (Fig. 2A). In the training chamber, mice were trained to initiate a trial by poking the center port, and the target and nontarget stimuli were presented simultaneously. Licking the water spout at the side of the positive stimulus was rewarded with water (5 μl), and licking at the incorrect side was followed by a timeout (10 s). We used training and visual acuity measurement modes similar to those in the water task.

To examine the efficiency of the new training chamber, we trained two groups of naive mice subjected to either a water maze or a customized training chamber. Approximately 80–120 trials can be executed every day in this training chamber, which is 4–6 times the trial number executed in the water maze. Therefore, fewer sessions were needed to obtain a high correct rate of the initial task learning (Fig. 2B). After 7 days of training in the chamber, the average VA of the mice showed a significant improvement, which was similar to that after 25 days of training in the water maze (chamber: Pre: 0.52 ± 0.01 cpd vs. Post: 0.79 ± 0.03 cpd, *p* < 0.005, water maze: Pre: 0.46 ± 0.01 cpd vs. Post: 0.71 ± 0.02 cpd, chamber, *n* = 12; water maze, *n* = 18, *p* < 0.005, Fig. 2C, Additional file 1: Fig. S1C).

Next, to confirm the involvement of the mPFC in this new VPL model, mice received either NMDA or sham lesions. Two groups of mice successfully acquired the visual discrimination task after the same training duration. No significant difference between the two groups was found in the trial number, reaction time (the time between the appearance of a visual stimulus and a response), correction (the percentage of the correct responses of all correct and incorrect responses), or accuracy (the percentage of the correct responses of all successfully triggered trials) of task execution (Additional file 1: Fig. S1D-G). The average VA after NSF training in the lesioned group increased to 0.64 ± 0.03 cpd, which was significantly lower than the 0.75 ± 0.02 cpd in the control group (Fig. 2D). Another group of trained mice was randomly divided into two groups, which were subjected to either bilateral mPFC lesions or sham-mPFC lesions. We observed a significant decrease in VA in the lesioned group and no significant change in VA in the sham group (lesion group: Post: 0.81 ± 0.03 cpd vs. Re: 0.56 ± 0.02 cpd, *p* < 0.05, sham group: Post: 0.82 ± 0.02 cpd vs. Re: 0.81 ± 0.02 cpd, NMDA, *n* = 5; sham, *n* = 5, *p* > 0.05, Fig. 2E). These findings indicated that the mPFC was necessary for the training and maintenance of VPL in the chamber. Therefore, this training chamber was used for subsequent calcium recording experiments.

### Inhibition of mPFC pyramidal neurons is sufficient to affect the VPL training process

**Fig. 3:**
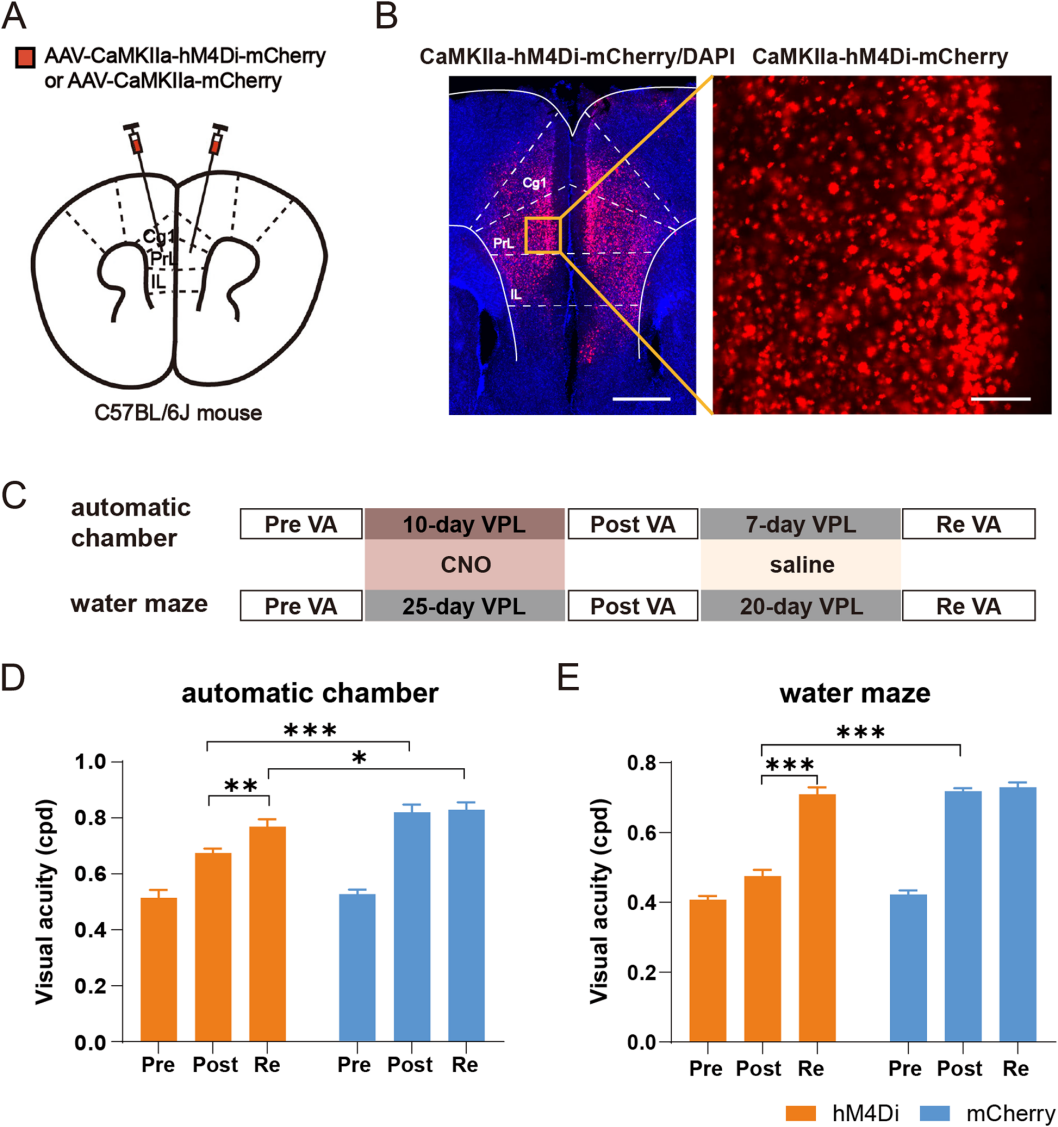
Chemogenetic inhibition of mPFC pyramidal neurons is sufficient to affect the process of VPL in two tasks. **A** A schematic of hM4Di or mCherry virus injection in the mPFC. **B** Left: a representative image of infected mPFC pyramidal neurons; scale bar: 500 μm. Right: enlarged view of CaMKIIa-hM4Di-mCherry-labeled pyramidal neurons in the prelimbic area indicated by the yellow box in the left figure; scale bar: 100 μm. **C** Top: schematic of the experimental procedure in the automatic chamber. Bottom: schematic of the experimental procedure in the water maze. **D** In the training chamber, the pretraining VA and VA after training with CNO and the retesting after training with saline were obtained from the hM4Di and mCherry groups, respectively. hM4Di: *n* = 6; mCherry: *n* = 7. **E** In the water maze, the pretraining VA and VA after training with CNO and the retesting after training with saline were obtained from the hM4Di and mCherry groups, respectively. hM4Di: *n* = 6; mCherry: *n* = 7. Data are represented as the mean ± SEM. ∗ , *p* < 0.05; ∗  ∗ , *p* < 0.01; ∗  ∗  ∗ , *p* < 0.005; n.s., not significant (*p* > 0.05)

To determine whether inhibiting the activities of mPFC pyramidal neurons is sufficient to affect the process of VPL in two VPL models, we reversibly inhibited the activity of mPFC pyramidal neurons with chemogenetic manipulation. We virally introduced the chemogenetic inhibitory DREADD hM4Di into the mPFC pyramidal neurons of C57BL/6 J adult male mice at postnatal day 60 (Fig. 3A–C). Mice injected with AAV2-mCherry were used as controls. Four weeks later, CNO was delivered to both the hM4Di and mCherry groups 30 min prior to the start of the behavior task in each session. When training in the chamber, the average VA after training in the hM4Di group with CNO application was 0.68 ± 0.02 cpd, which was significantly lower than the 0.82 ± 0.03 cpd in the mCherry group. After removing chemogenetic inhibition, the VA of the hM4Di group significantly increased to a similar level as that in the control group (Fig. 3D). Consistent results were also shown in the training of the visual water task (Fig. 3E). The overall results indicate that the activity of mPFC pyramidal neurons is important for VPL.

### Distinct mPFC pyramidal neuron ensembles differently tuned to poking or reward behaviors

**Fig. 4:**
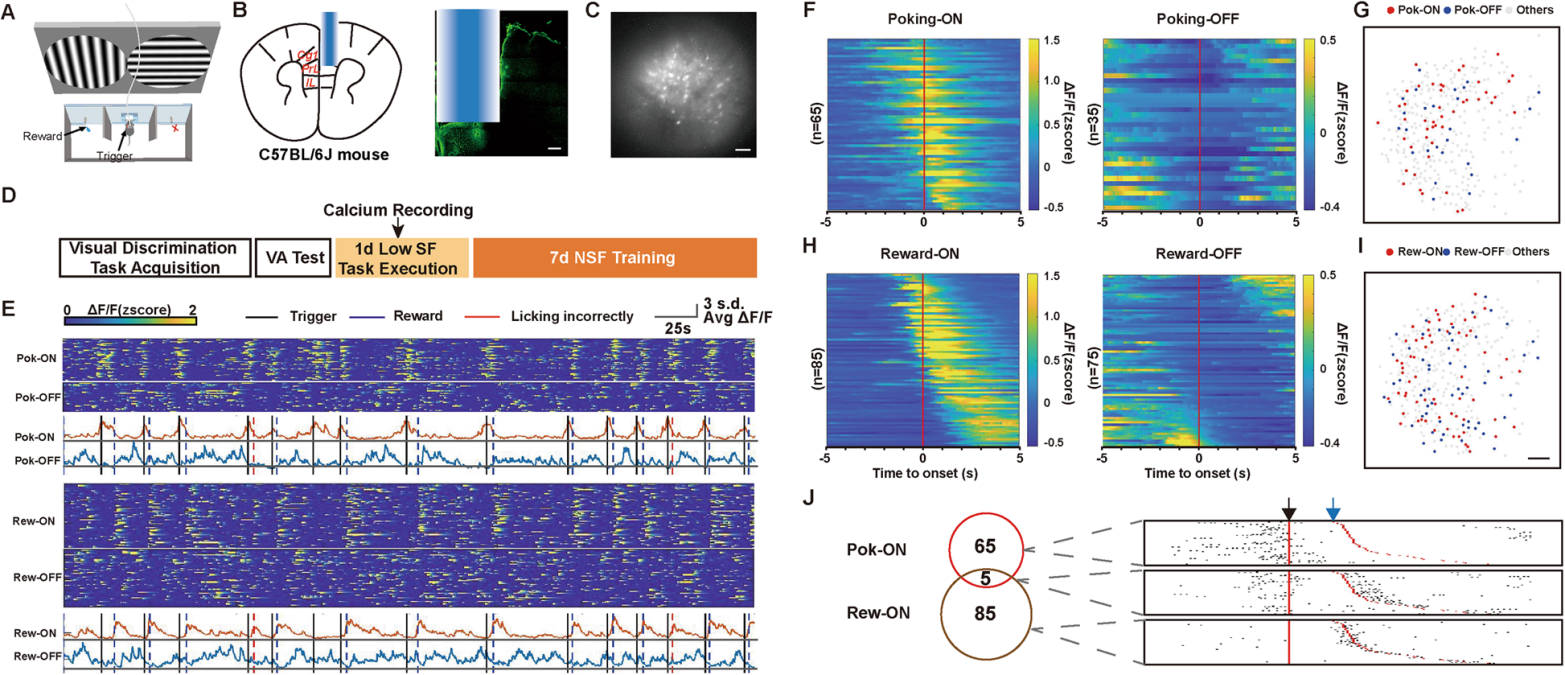
Calcium activities of mPFC pyramidal neuron ensembles tuned to specific task-related behaviors. **A** Diagram of calcium recording in the custom-designed training chamber. **B** Left: a schematic of GRIN lens implantation in the mPFC. Right: a representative image of injection with GCaMP6f and implantation of a GRIN lens in the mPFC. Scale bar: 200 μm. **C** A representative image of mPFC pyramidal neurons labeled with GCaMP6f at maximum projection fluorescence. Scale bar: 100 μm. **D** A schedule of calcium recording. **E** Raster plots of individual neural calcium activities and calcium traces of the averaged group activity of Poking-ON, Poking-OFF, Reward-ON, and Reward-OFF neurons (Pok-ON, Pok-OFF, Rew-ON, and Rew-OFF) from a representative mouse. Vertical black lines, onset of poking; vertical blue-dotted lines, onset of reward; vertical red-dotted lines, onset of licking behavior without water reward. **F** Raster plot of the averaged *z* score of individual Poking-ON and Poking-OFF neurons at the onset of poking behavior (5 s before and 5 s after), sorted by the time of maximal activities (ON neurons) or minimal activities (OFF neurons). **G** Spatial distributions of Pok-ON and Pok-OFF neurons from the same representative mouse; scale bar, 100 μm. **H** Raster plot of the averaged *z* score of individual Reward-ON and Reward-OFF neurons at the onset of drinking behavior (5 s before and 5 s after), sorted by the time of maximal activities (ON neurons) or minimal activities (OFF neurons). **I** Spatial distributions of Rew-ON and Rew-OFF neurons from the same representative mouse; scale bar, 100 μm. **J** Left: schematic of interbehavior overlap between Pok-ON and Rew-ON neural ensembles. Right: calcium events per trial aligned to poking behavior onset (black arrow) are shown from an example Pok-ON neuron (top), an example Pok-ON and Rew-ON neuron (middle) and an example Rew-ON neuron (bottom). The blue arrow shows the onset of drinking behavior

To identify how mPFC pyramidal neurons encoded information about attention or reward, we then synchronously recorded mouse behavior and calcium activities of mPFC pyramidal neurons when mice executed the visual discrimination task (Fig. 4A–D). We first examined whether carrying a miniature fluorescence microscope (miniScope) influenced the task performance of mice. After mice successfully acquired the visual discrimination task, the correction (the percentage of the correct responses of all correct and incorrect responses), accuracy (the percentage of the correct responses of all successfully triggered trials), and reaction time (the time between the appearance of a visual stimulus and a response) of task execution were not significantly different between the miniScope group and the control group (Additional file 1: Fig. S2A-C). We manually annotated two behaviors that might carry information about attention and reward: “Poking” when mice poke the center port to initiate a new trial with impending water-persuing behavior and “Reward” when mice lick at the correct side to obtain the water reward. Calcium transients from mPFC excitatory neurons were detected and extracted by an extended constrained nonnegative matrix factorization (CNMF-E) framework [34]. We recorded 521 excitatory neurons from 5 mice at this recording stage. Similar to previous studies [35–37], we defined mPFC neurons specifically tuning to different behaviors by calculating each neuron’s observed calcium-behavior similarity and comparing it with its own chance level of calcium-behavior similarity (Methods; Additional file 1: Fig. S2D, S2E). Both ON and OFF neuronal groups were identified to tune to “Poking” and “Reward” behaviors (Poking-ON, Poking-OFF, Reward-ON and Reward-OFF neurons) (Fig. 4E). The averaged calcium activity for each ON and OFF neuron across all behavioral epochs was calculated and sorted based on the time of their peak and minimal activities, respectively (Fig. 4F, H). ON and OFF neurons tuned to each specific behavior were mutually exclusive and spatially scattered (Moran’s I for each ensemble in each mouse, *p* > 0.05, Fig. 4G, I). These results indicate the existence of specialized ON and OFF neural ensembles in mPFC tuning to specific real-time behavioral information with opposing activities.

We focused on ON neurons in the following study, as relatively weak responses of OFF neurons tuned to both behaviors were observed (Additional file 1: Fig. S2F, S2G). In different trials, the time interval between poking and drinking varied. Thus, we aligned the onset of poking (black) and sorted the calcium events per trial based on the time interval between poking and drinking (blue). Three example ON neurons are shown in Fig. 4J. The Poking-ON and Reward-ON ensembles were largely nonoverlapping (Fig. 4J), suggesting that most neurons show “ON” responses specifically to one behavior.

**Fig. 5:**
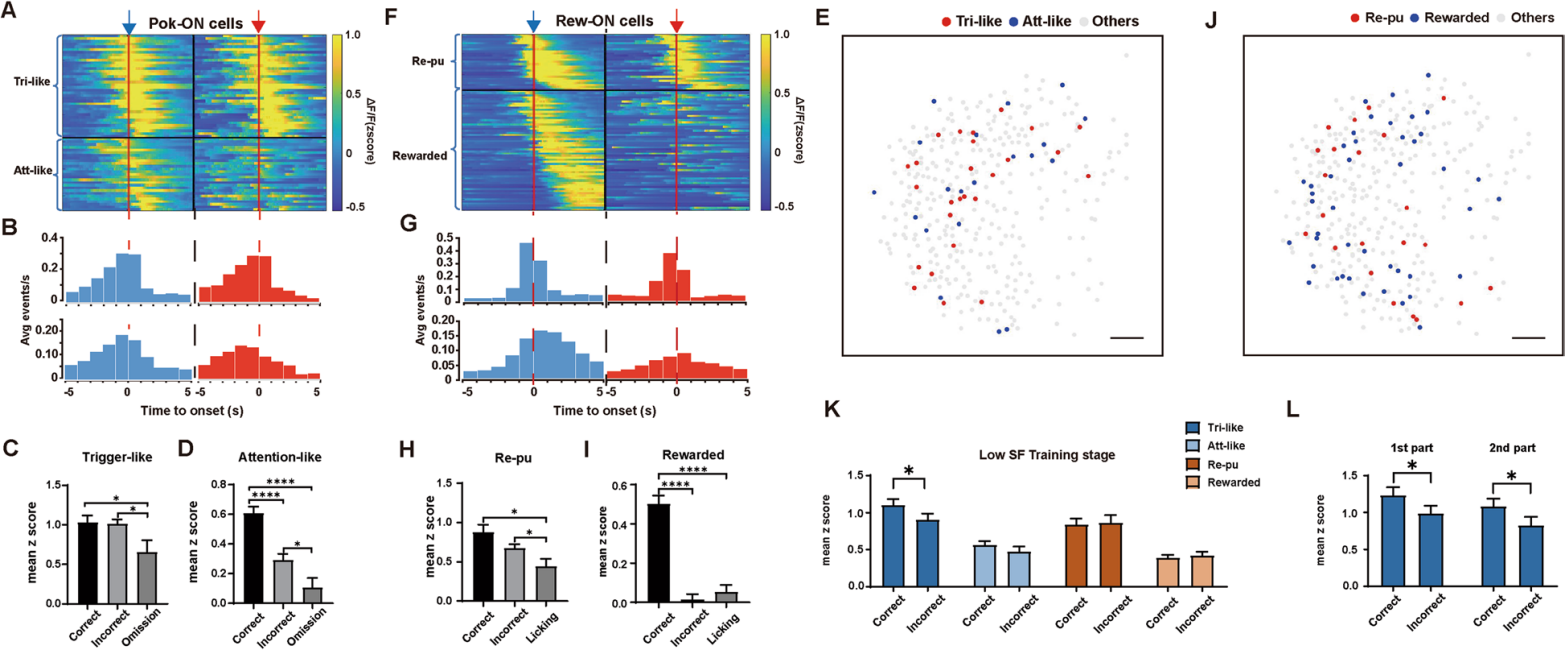
Distinct mPFC pyramidal neuron ensembles differently tuned to poking or reward behaviors. **A** Raster plot of the averaged *z* score at the onset (red line) of poking behavior (5 s before and 5 s after) of individual “Trigger-like” (Tri-like) and “Attention-like” (Att-like) neurons in correct (blue arrow) and incorrect (red arrow) trials, sorted by the time of maximal activities in correct trials. **B** Histograms of calcium events aligned to poking behavior onset (red-dotted line) of “Trigger-like” (top) and “Attention-like” (bottom) groups in correct (left) and incorrect (right) trials. **C** The mean *z* score during the Poking time window on correct, incorrect, and omission trials of the “Trigger-like” group. *n* = 38. **D** The mean *z* score during the Poking time window on correct, incorrect, and omission trials of the “Attention-like” group. *n* = 27. **E** Spatial distributions of “Trigger-like” and “Attention-like” neurons from the same representative mouse; scale bar, 100 μm. **F** Raster plot of the averaged *z* score at the onset (red line) of licking behaviors (5 s before and 5 s after) of individual “Reward-pursuing” (Re-pu) and “Rewarded” neurons in correct (blue arrow) and incorrect (red arrow) trials, sorted by the time of maximal activities in correct trials. **G** Histograms of calcium events aligned to reward onset (red-dotted line) of “Reward-pursuing” (top) and “Rewarded” (bottom) groups in correct (left) and incorrect (right) trials. **H** The mean *z* score during the reward time window on correct, incorrect, and licking trials of the “Reward-pursuing” group. *n* = 27. **I** The mean *z* score during the reward time window on correct, incorrect, and licking trials of the “Rewarded” group. *n* = 58. **J** Spatial distributions of “Reward-pursuing and “Rewarded” neurons from the same representative mouse; scale bar, 100 μm. **K** Neuronal response of each ensemble modulated by previous outcomes. Tri-like: *n* = 38; Att-like: *n* = 27; Re-pu: *n* = 27; Rewarded: *n* = 58. **L** Neuronal response of the “Trigger-like” ensemble modulated by previous outcomes at two parts of the low SF execution stage. *n* = 38. Data are represented as the mean ± SEM. ∗ *p* < 0.05; ∗  ∗ *p* < 0.01; ∗  ∗  ∗ *p* < 0.005; ∗  ∗  ∗  ∗ *p* < 0.001; n.s., not significant (*p* > 0.05)

When mice actively execute the visual discrimination task, many high-level cognitive functions may contribute to different behaviors. Attention, the motivation that drives trigger behavior, motor preparation, and response to visual stimuli, may play a role in the poking window. In the reward window, neural responses can be affected by many other cognitive components, such as hidden state inference, prospective encoding of outcome, and reward detection. To distinguish how mPFC pyramidal neurons encode different information, we further classified Poking-ON and Reward-ON neurons into different subsets according to their different response characters in correct and incorrect trials. Forty-two percent Poking-ON neurons (27/65) showing “ON” responses only in correct trials were identified in our study and were defined as “Attention-like” neurons due to their similar response character as the attention neuron in previous electrophysiological study [18]. Other Poking-ON neurons (38/65) showing “ON” responses in both correct and incorrect trials were defined as “Trigger-like” neurons (Fig. 5A). Histograms of calcium event distribution indicated that both “Trigger-like” and “Attention-like” neurons fired mostly before poking onset (Fig. 5B). To further examine whether the ON response of “Trigger-like” neurons is related to motor preparation for the trigger behavior, we compared the mean amplitudes of neural response in correct and incorrect trials with the omission trials, in which animals only poked the nose to initiate the trial without following reward pursuing behavior. Responses in omission trials were mild but significantly lower than those in either correct (correct: 1.03 ± 0.09 vs. omission: 0.65 ± 0.15, *p* < 0.05, one-way ANOVA followed by Tukey’s multiple-comparisons test) or incorrect trials (incorrect: 1.01 ± 0.06, *p* < 0.05; one-way ANOVA followed by Tukey’s multiple-comparisons test, Fig. 5C). These results indicated that both motivation and motor preparation might play a role in the response of “Trigger-like” neurons. As expected, responses in omission trials of the “Attention-like” ensemble were significantly lower than those in incorrect trials of the “Attention-like” ensemble (incorrect: 0.29 ± 0.04 vs. omission: 0.11 ± 0.06, *p* < 0.05, one-way ANOVA followed by Tukey’s multiple-comparisons test, Fig. 5D) and had no significant difference from the baseline response (*p* > 0.05), suggesting that motivation and motor preparation had minor effects on the response of “Attention-like” neurons. Last, mPFC neurons responding to visual stimuli would show an “ON” response only after poking onset regardless of the outcome. Only four neurons with this characteristic were identified in the “Trigger-like” ensemble (Additional file 1: Fig. S3A, S3B). Both “Trigger-like” and “Attention-like” neurons were distributed scatteredly in the mPFC (Moran’s I for each ensemble in each mouse, *p* > 0.05, Fig. 5E).

We also divided Reward-ON neurons into two subsets with different response properties to different outcomes and examined their responses in licking trials in which mice failed to obtain the reward by licking the spout without initiating the trial. “Reward-pursuing” neurons (27/85) showed strong calcium responses in both correct and incorrect trials (correct: 0.88 ± 0.10 vs. incorrect: 0.67 ± 0.05, *p* = 0.092, one-way ANOVA followed by Tukey’s multiple-comparisons test, Fig. 5H) and mild but significantly lower responses in licking trials (licking: 0.44 ± 0.09, *p* < 0.05, one-way ANOVA followed by Tukey’s multiple-comparisons test, Fig. 5H). The calcium events of “Reward-pursuing” neurons were transient and mostly distributed around the onset of licking behavior. We therefore supposed that the activities of “Reward-pursuing” neurons were related to hidden state inference. “Rewarded” neurons (58/85) showed an “ON” response only when the outcome was a water reward (correct: 0.50 ± 0.04 vs. incorrect: 0.014 ± 0.029, *p* < 0.001, one-way ANOVA followed by Tukey’s multiple-comparisons test, Fig. 5I). Most “Rewarded” neurons showed sustained calcium activity after the mice were rewarded (Fig. 5G). Responses of “Rewarded” neurons in licking trials were not significantly different from those in incorrect trials (licking: 0.054 ± 0.036, *p* = 0.26, one-way ANOVA followed by Tukey’s multiple-comparisons test, Fig. 5I) and were distinct from those of “Reward-pursuing” neurons in licking trials (*p* = 1.08E^−05^). Moreover, responses in incorrect and licking trials were both similar to the baseline level (*p* > 0.05). These findings suggested that activities in “Rewarded” neurons might be driven by reward detection and consummatory behavior. Neurons of both groups were randomly distributed in the mPFC (Moran’s I for each ensemble in each mouse, *p* > 0.05, Fig. 5J). Previous study reported a few neurons in rat mPFC showing location preference [44]. To investigate if the response of “Rewarded” neurons is affected by different port location (right or left), we also compare the response difference at different port location of each Rewarded neurons. In the present study, some Rewarded neurons did show significantly stronger response to either left or right port, while more than half of which showing no port location bias (Additional file 1: Fig. S4).

To investigate whether reward experience in the last trial modulates subsequent neuronal activities, we grouped trials according to the animal’s previous outcomes (correct or incorrect) and compared the neuronal responses of four distinct neuronal ensembles. Trigger-like neurons exhibited significantly higher responses after correct trials (after correct trials: 1.10 ± 0.08 vs. after incorrect trials: 0.90 ± 0.09, *p* < 0.05, Fig. 5K), while no significant difference was found in the other three groups. We supposed that the activity selectivity of Trigger-like neurons was related to the different motivation intensities induced by different previous behavioral outcomes. To explore whether this response difference of Trigger-like neurons would disappear after mice acquired a certain amount of water, we compared the Trigger-like neuron response in the first and second half of training trials. Similarly, significantly higher responses after correct trials were recorded in both halves of the training (Fig. 5L). Therefore, this response difference may not be affected by the state of mouse thirst. Collectively, we identified distinct poking- or reward-related ensembles with different response characteristics at the low SF execution stage. The reward requirement may modulate the subsequent activities of Trigger-like neurons at this recording stage.

### More reward-related components participate in the task after VPL training

**Fig. 6:**
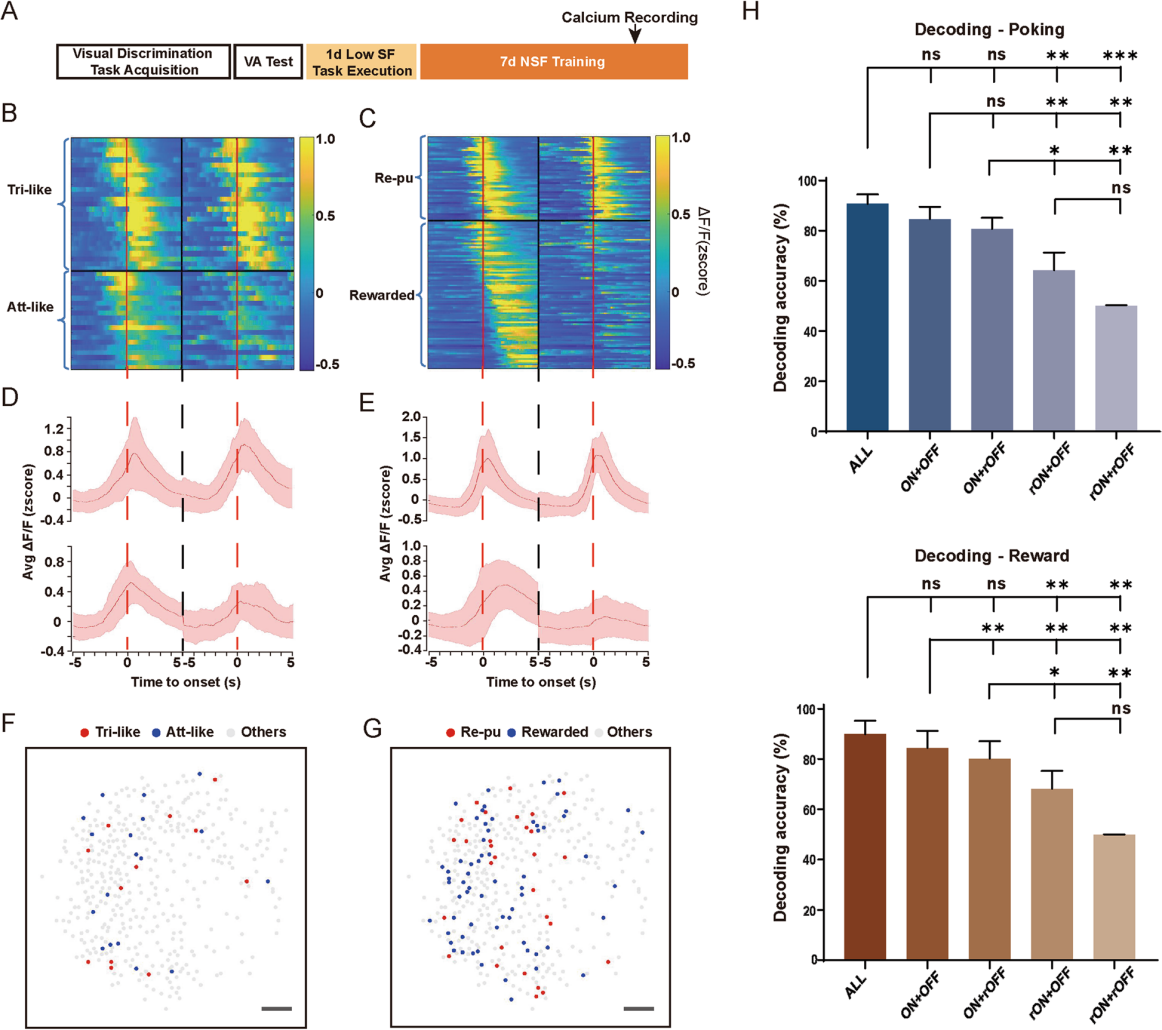
Neuronal ensembles during the NSF training stage. **A** A schedule of calcium recording. **B** Raster plot of the averaged *z* score at the onset of poking behavior (5 s before and 5 s after) of individual “Trigger-like” and “Attention-like” neurons in correct and incorrect trials, sorted by the time of maximal activities in correct trials. **C** Raster plot of the averaged *z* score at the onset of licking behavior (5 s before and 5 s after) of individual “Reward-pursuing” and “Rewarded” neurons in correct and incorrect trials, sorted by the time of maximal activities in correct trials. **D** Plots show the averaged group calcium activity of correct (left) and incorrect (right) trials at the onset (red-dotted line) of poking behavior (± 5 s) of “Trigger-like” (top) and “Attention-like” (bottom) neurons. **E** Plots show the averaged group calcium activity of correct (left) and incorrect (right) trials at the onset (red-dotted line) of licking behavior (± 5 s) of “Reward-pursuing” (top) and “Rewarded” (bottom) neurons. **F** Spatial distributions of “Trigger-like” and “Attention-like” neurons from the same representative mouse; scale bar, 100 μm. **G** Spatial distributions of “Reward-pursuing” and “Rewarded” neurons from the same representative mouse; scale bar, 100 μm. **H** Decoding analyses for poking (top) and reward (bottom) behavior using calcium activity from different groups of neurons. ALL, all imaged neurons; ON + OFF, ON and OFF neurons together; ON + rOFF, ON and shuffled OFF neurons; rON + OFF, OFF and shuffled ON neurons; rON + rOFF, shuffled ON and OFF neurons. *n* = 5. Data are represented as the mean ± SEM. ∗ , *p* < 0.05; ∗  ∗ , *p* < 0.01; ∗  ∗  ∗ , *p* < 0.005; n.s., not significant (*p* > 0.05)

To examine how VPL training modulated the activity selectivity of mPFC pyramidal neurons in favor of behavioral needs, we recorded calcium activity on the fifth NSF training day when the VA of mice increased above 40% (Fig. 6A). We recorded 617 excitatory neurons in the mPFC of the same mice, suggesting that VPL training might recruit more mPFC neurons. At the NSF training stage, similar “ON” and “OFF” neurons in the mPFC tuned to poking and successful water licking behaviors were also recorded (Additional file 1: Fig. S5), and the two “ON” ensembles could be further divided into “Trigger-like,” “Attention-like,” “Reward-pursuing,” and “Rewarded” groups (Figs. 6B–D). These neural ensembles showed similar response properties to those before the training (Additional file 1: Fig. S4, S6). All neuron ensembles were spatially scattered and intermixed (Moran’s I for each ensemble in each mouse, *p* > 0.05, Fig. 6F,G). We then performed decoding analyses to examine whether different behaviors could be predicted by activities of ON and OFF neural ensembles. We found that decoding performance using the population responses of the ON neural ensemble only was similar to those using ON and OFF neural ensembles together or all imaged neurons (*p* > 0.05; one-way ANOVA followed by Tukey’s multiple-comparisons test, Fig. 6H). To exclude the potential effect of different sampling size of ON and OFF ensembles, we re-calculated the decoding accuracy after balancing the neuron number in two groups (randomly selected the same number of ON neuron with OFF neurons for ten times). A significantly higher accuracy was still acquired in ON ensemble (Additional file 1: Fig. S7). We therefore presume the relatively low decoding accuracy predicted by OFF neural ensemble possibly due to the relatively weak response of OFF neurons (*p* < 0.001; one-way ANOVA followed by Tukey’s multiple-comparisons test).

**Fig. 7:**
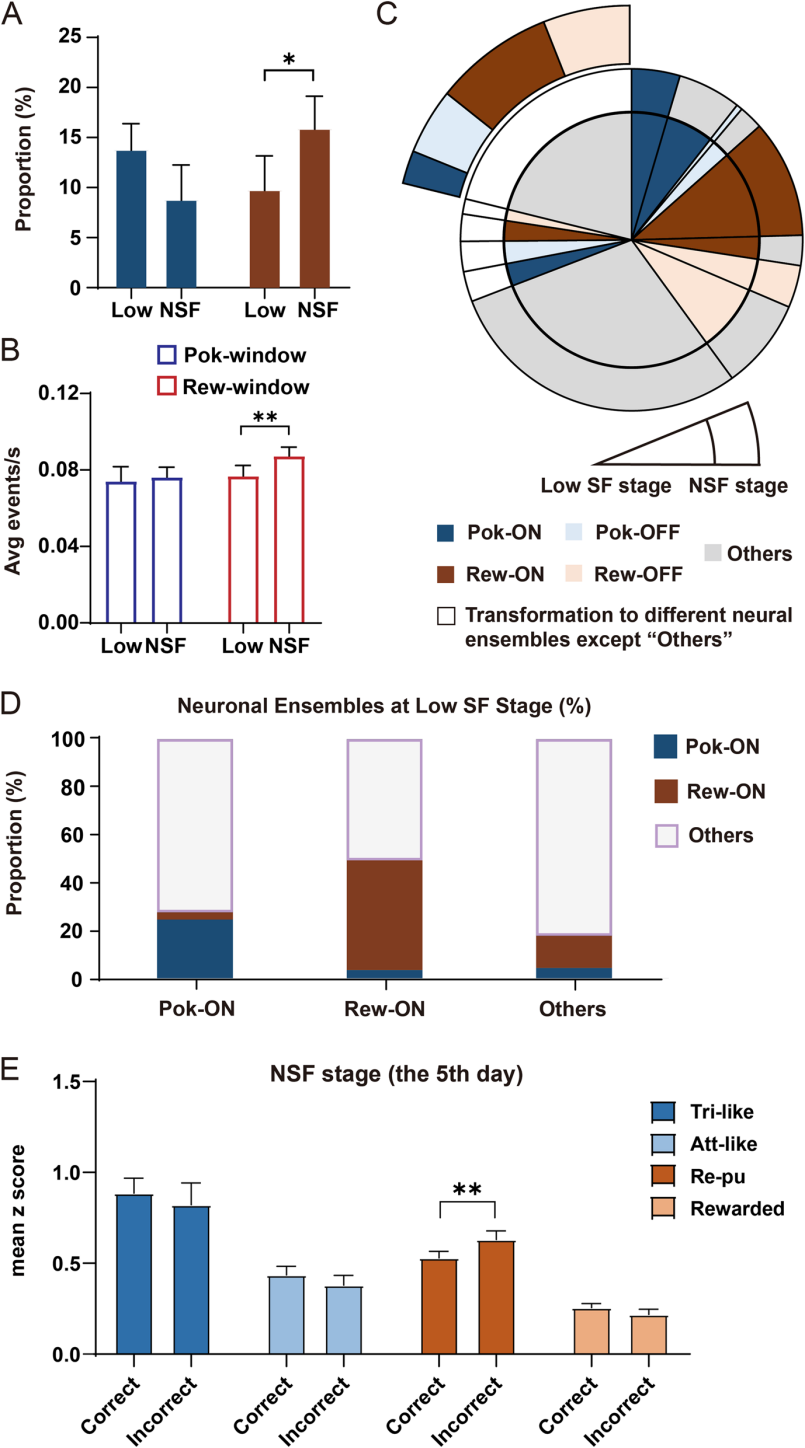
More Reward-ON-related components participate in the task after VPL training. **A** In the tracked neurons, the proportions of Pok-ON and Rew-ON neurons at the low SF execution stage and high SF NSF training stage. *n* = 5. **B** The average calcium event frequency of the tracked neurons at poking-related time windows (Pok-window) and reward-related time windows (Rew-window). *n* = 350. **C** The transform proportion between the two stages of the tracked neurons. **D** Proportion of Pok-ON, Rew-ON, and Other neurons at the NSF training stage among Pok-ON, Rew-ON, and Other neurons at the low SF stage. *n* = 788 neurons. **E** Neuronal response of each ensemble at the corresponding time window after different feedbacks from the previous trials at the fifth NSF training day. Tri-like: *n* = 27; Att-like: *n* = 20; Re-pu: *n* = 46; Rewarded: *n* = 81. Data are represented as the mean ± SEM. ∗ , *p* < 0.05; ∗  ∗ , *p* < 0.01; ∗  ∗  ∗ , *p* < 0.005; n.s., not significant (*p* > 0.05)

Neural populations at the two different stages were not identical, but they shared approximately 67% (350/521) common neurons (Additional file 1: Fig. S8). We defined the subset of neurons identified across the two different stages as the “tracked neurons”. In the tracked neurons, the proportion of the Reward-ON ensemble significantly increased at the NSF training stage (low SF stage: 9.9% ± 3.3% vs. NSF training stage: 16.2% ± 3.2%, *n* = 5, *p* < 0.05), while the Poking-ON ensemble relatively decreased (low SF stage: 13.9% ± 2.5% vs. NSF training stage: 8.9% ± 3.4%, *n* = 5, *p* = 0.093, Fig. 7A). To exclude the possibility that these changes were due to the high SF task used in the NSF training stage, we calculated the proportion of each neural ensemble in the whole neuronal population on the first NSF training day when mice were also trained with a high SF task. The proportion distribution of neural ensembles on the first NSF training day was similar to that at the low SF task execution stage, rather than that at the NSF stage, indicating that the change was due to VPL training (Additional file 1: Fig. S9A). For the tracked neurons, we also calculated the average calcium event frequency at each behavioral time window at two different stages. During the reward-related time window, calcium event frequency significantly increased at the NSF training stage, while no significant difference was found at the poking-related time window (Poking window: low SF stage: 0.076 ± 0.006 vs. NSF training stage: 0.077 ± 0.006, *n* = 350, *p* > 0.05, Reward window: low SF stage: 0.076 ± 0.005 vs. NSF training stage: 0.087 ± 0.005, *n* = 350, *p* < 0.001, Fig. 7B).

A previous study showed that neural ensembles tuned to direct exploration in the mPFC are highly dynamic [37]. Thereafter, we examine the transformation of individual neurons in the tracked neurons. More than 90% of neurons were still in the same group or switched between one of the behavioral-related ensembles and the “Other” neural ensemble. In particular, transformation between Poking-ON and Reward-ON ensembles was relatively rare (Fig. 7C, Additional file 1: Fig. S9B). We next split each recording day into two halves and reidentified ON and OFF neurons tuned to distinct behaviors to investigate how cells switch classifications at the same stage. More than 98% of neurons were identified to be in the same group or switched between one of the behavioral-related ensembles and the “Other” neural ensemble at each recording stage (Additional file 1: Fig. S9C). These results indicated subtle shifts in the tuning properties of neural ensembles after a 5-day training. To examine the neural coding stability across two different stages, we pooled data together from the low SF stage and the NSF training stage. Nearly half of Reward-ON neurons (45.9%) identified at the low SF stage continued coding the reward information after 5 days of VPL training, while only 24.6% of Poking-ON neurons coded the poking information. Consistently, more Reward-ON neurons (13.5%) were recruited than Poking-On neurons (4.4%) from the “Other” ensemble after VPL (Fig. 7D). Therefore, fewer Poking-ON and more Reward-ON neurons were recruited at the NSF training stage.

We also explored how previous outcomes modulate subsequent neuronal activities after a period of VPL learning. Different from that at the low SF execution stage, Trigger-like neurons at the NSF training stage no longer showed a significant previous outcome selectivity. “Attention-like” and “Rewarded” neurons retained similar responses regardless of whether a previous trial was succussed. In “Reward-pursuing” neurons, however, significantly higher responses after previous incorrect trials emerged after VPL learning (after correct trials: 0.53 ± 0.04 vs. after incorrect trials: 0.63 ± 0.05, *p* < 0.001, Fig. 7E), suggesting that hidden state inference might play an important part in driving VPL. Therefore, after NSF VPL training for a period of time, reward information might modulate neuronal activities in a different way, with a significant previous outcome selectivity shifting from the Trigger-like ensemble to the Reward-pursuing ensemble. The overall results indicate that four subdivided behavior-related “ON” ensembles stably existed at the NSF training stage, while more reward-related components were involved in the VPL task after NSF VPL training for a period of time.

## Discussion

In this study, we proved that the mPFC played an important role in VPL. By recording calcium activities in free-moving mice in a customized training chamber, we further showed that mPFC pyramidal neurons formed distinct ensembles preferentially tuned to different task-related behaviors throughout the training process and that more Reward-ON neurons were activated during visual perceptual training.

### The mPFC plays an important role in the VPL model

Our findings in mice provide the first direct evidence that the mPFC is involved in the process of VPL. Bilateral lesion and chemogenetic inhibition of the mouse mPFC greatly impaired the VA improvement induced by VPL and the maintenance of perceptual learning. Our results support human fMRI studies that show correlated activation of prefrontal regions with perceptual training [15–17]. However, inactivation of the mPFC cannot completely block vision improvement by VPL, indicating that other brain areas also participate in the process of VPL. The mouse mPFC was found to strongly influence sensory processing in the primary visual cortex (V1) [38, 39]. Meanwhile, a resting-state fMRI study in humans showed that functional connectivity between the visual cortex and prefrontal association areas was related to visual discrimination task performance [17]. Many studies, including ours, have reported functional and structural changes in V1 associated with task-relevant VPL [8, 12–14]. Previous studies proposed the possibility that top-down influence or feedback from higher cortical areas modulates VPL-related V1 activation changes [40, 41]. We therefore proposed that the mPFC, V1, and the top-down modulation between them may be critically involved in the process of VPL.

Our study also demonstrated that pyramidal neuronal ensembles positively and negatively correlated with different behavioral events in the visual perceptual training task coexisted in the mPFC. Meanwhile, ON and OFF ensembles tuned to the same behavior were spatially intermingled (Fig. 3G, I; Additional file 1: Fig. S2F). Most calcium imaging studies carried out in the mPFC focused on task-related neural ensembles with enhanced activities [33, 42, 43], while only a few studies have demonstrated the coexistence of behaviorally tuned ON and OFF ensembles among mPFC excitatory neurons [37]. Different from the similar response intensity of ON and OFF ensembles recorded in social behavioral tests, we recorded relatively weak responses of OFF ensembles compared with ON ensembles, which is similar to a previous electrophysiological study [18]. We proposed that the relative intensity between ON and OFF ensembles might be due to different types of behaviors. The relatively weak responses of OFF ensembles might lead to the lower decoding accuracy of OFF ensembles compared with ON ensembles (Fig. 6H).

### More reward-related components were involved in the NSF training stage

In the present study, we designed a novel VPL training chamber based on reward-driven goal-directed behavior. We first identified ON neuronal ensembles toward poking and reward behaviors at the low SF task execution stage. Similar to previous studies, we further identified “Attention-like” neurons from the Poking-ON group showing larger responses in correct trials, a similar response property to a group of neurons that was related to the level of preparatory attention during an attention task [18]. The response characteristic of “Reward-pursuing” neurons identified from the Reward-ON group is consistent with previous electrophysiological findings in the rat mPFC that some neurons fire immediately prior to reaching the reward zone without discrimination between the correct and incorrect conditions [45, 46]. We supposed these neurons encoding information about hidden state inference. Similarly, the “Rewarded” ensemble identified from the Reward-ON group showed both transient and sustained calcium signals, which are similar to those reported in primate PFC neurons in different electrophysiological findings [47–49]. Previous study showed a few location preference neurons in rat mPFC [44]. We also investigated if different port location (right or left) affects the response of “Rewarded” neurons. In the present study, some Rewarded neurons did show significantly stronger response to either left or right port, while more than half of which showing no port location bias (Additional file 1: Fig. S4). “Trigger-like” neurons that fired before poking behavior onset may encode motivation. A previous study demonstrated that the circuits of the PFC regulate motivation [19]. However, neuronal activity related to this cognitive component was not reported in previous studies. Our present study provides evidence at the cellular level for the first time. At the low SF task execution stage, we also found that Trigger-like neurons showed significantly higher responses after correct trials that delivered water rewards. Reward was thought to influence attention indirectly by modulating task-specific motivation in previous studies [50], and our present study may provide the first evidence at the cellular level in the mouse mPFC.

In the NSF training stage, we found that a smaller proportion of Poking-ON neurons and more reward-related components were involved in the task. Both the increased calcium activity during the reward-related time window and more activated neurons recorded at the NSF training stage in our study might support a previous study that an enhanced BOLD signal in the human DLPFC is correlated with the amount of perceptual learning [51]. Many VPL models, including ours, are reward-driven and rely on goal-directed processes. Although a few studies have shown that training improvements can occur even without reward in a range of task conditions [52], more studies have shown that reward plays a critical role in experience-dependent adult cortical plasticity [53] and promotes visual perceptual learning (VPL) [54–56]. A previous study in humans found that high reward can greatly improve the magnitude of perceptual learning [57]. Previous studies also indicated that the mPFC plays a role in reward-related mechanisms such as prediction or evaluation of rewards by its dopamine innervation [58, 59]. Above all, the reward-related projection in mPFC might play an important role in VPL. Our results provide direct evidence for the involvement of reward-related components in mPFC during perceptual learning process. Both attention and reward contribute to some forms of perceptual learning [60]. Our study could not exclude the possibility that attention is important to VPL. A negative correlation between activations in some attention-related areas and the degree of perceptual learning was revealed in a previous study [15]. The less activated Poking-ON neurons in our study might support this phenomenon.

Previous rewards could influence animal behavior in subsequent trials [61]. However, little is known about which brain areas participate in this process and their related neural activities. In our present study, we first proposed that the “Trigger-like” and “Reward-pursuing” ensembles in the mouse mPFC might link prior reward to neuronal activities in subsequent trials at the visual discrimination task stage and VPL stage, respectively. At the NSF VPL training stage, Trigger-like neurons no longer showed a significant response difference after correct and incorrect trials, while the Reward-pursuing ensemble showed significantly higher neuronal responses after incorrect trials than after correct trials. Individual neurons in the monkey DLPFC were found in previous studies encoding signals related to their choice and its outcome in the previous trial [47, 48] and might impact the process animals seeking an optimal decision-making strategy according to a reinforcement learning algorithm. Therefore, the change in neuronal ensembles related to the outcome in the previous trial in our present study may suggest a different neuronal encoding mechanism in the VPL process. However, the exact mechanism by which those signals are involved in the VPL process to update the value information or decision-making strategies is not known. More future research is required to better understand the relationships between different cognitive components and perceptual learning.

### Dynamic population coding model

In the present study, we examined neural coding stability across two different stages. Neural ensembles identified to tune to the same behavior were not identical at different stages, but they shared a group of common neurons. Relatively high dynamics of mPFC neural ensembles tuned to direct exploration behavior were shown in a previous study [37]. In our study, we found a much higher degree of ensemble overlap across different stages, suggesting relatively low dynamics of the behavior-related ensembles. We also found a relatively rare transformation between Poking-ON and Reward-ON ensembles, which might be controlled by specific rules to define boundaries for distinct neural ensembles. Our results supported the dynamic population coding model in the mPFC [37, 62]. We proposed that the dynamic difference might be due to the different task requirements. According to the limited research available thus far, relatively higher stability was reported in more sensation- or action-associated areas, while lower stability in the hippocampus was reported [63–67]. In our VPL task, the mouse mPFC may play a part in improving the ability to read out sensory information. We therefore propose that the relatively higher stability of mPFC neuronal representations found in our present study might be correlated with the connection with the visual cortex.

## Conclusions

In summary, we demonstrated that the mPFC was essential for both the training and maintenance processes of VPL in mouse models and revealed the neural mechanism underlying vision improvement following VPL and identify distinct ON and OFF neural ensembles in the mPFC that tuned to different information during visual perceptual training. Our findings suggest an important role of the mPFC in VPL, with more reward-related components being also involved, and pave the way for future clarification of the reward signal coding rules in VPL.

## Methods

### Key resources table

**Table:** 

REAGENT or RESOURCE	SOURCE	IDENTIFIER
Bacterial and Virus Strains		
AAV9-CaMKlla-Gcamp6f	Vigene China	N/A
AAV2/9-CaMKIIa-hM4Di-mCherry	Vigene China	N/A
AAV2/9-CaMKIIa-mCherry	Vigene China	N/A
Experimental Models: Organisms/Strains		
C57BL/6 J Mice	Vital River	Cat# 213
Software and Algorithms		
MATLAB R2021a	MathWorks	https://www.mathworks.com/downloads/ web_downloads/
ImageJ	National Institutes of Health	https://imagej.nih.gov/ij/index.html
GraphPad Prism	GraphPad	https://www.graphpad.com/
CNMF MATLAB toolbox	Pnevmatikakis et al. (2016)	https://github.com/epnev/ca_source_ extraction
Software and Algorithms	This work	N/A
Other		
Miniature microscope for in vivo calcium imaging	Barbera et al. (2016)	https://github.com/giovannibarbera/ miniscope_v1.0
GRIN lens	Go! Foton, USA	ILW-100-P0460-055-NC

### Subject details

All animal procedures were approved by the Institutional Animal Care and Use Committees of University of Science and Technology of China (approval number USTCACUC1901011) and performed in accordance with National Institute of Health guidelines. Male C57BL/6 J mice at 3–4 months of age and 25–35 g of weight were used in this study. All animals were group housed (maximum five mice per cage) under standard laboratory conditions with a 12/12 light–dark cycle (light from 8:00 A.M. to 8:00 P.M.), 21 °C ambient temperature and 35% relative humidity and were given food and water ad libitum. During the behavioral training stage, mice were deprived of water in the home cage and obtained a water reward during daily behavior sessions. On days that mice did not perform the task, restricted water access (~ 1 ml) was provided each day.

### NMDA/virus injection

All surgical tools and materials were sterilized with alcohol, and all procedures applied were aseptic. Mice were anesthetized with sodium pentobarbital/xylazine (sodium pentobarbital: 50 mg/kg; xylazine: 30 mg/kg, i.p.), maintained with 1–2% isoflurane, and placed in a stereotaxic frame. Body temperature was continuously monitored and maintained at 37 °C.

Excitotoxic lesions were induced through the injection of the neurotoxic drug N-methyl-D-aspartic acid (NMDA). For lesions within the mPFC, male C57BL/6 J mice received bilateral injection of NMDA (500 nl, 20 mg/L, Sigma) or phosphate-buffered saline (PBS) (0.1 M, pH = 7.4) at the following sites: A/P, + 1.95 mm; M/L, ± 0.40 mm from bregma; D/V, 1.40 mm from the dura. NMDA solution was freshly prepared on the day of surgery.

For manipulation of pyramidal neurons, 300 nL of CaMKIIa-hM4Di-mCherry (1.40 × 10^14^ genome copies/mL, Vigene, China) and CaMKIIa-mCherry (9.74 × 10^13^ genome copies/mL, Vigene, China) was delivered bilaterally to the mPFC of male C57BL/6 J mice (A/P, + 1.95 mm; M/L, ± 0.40 mm from bregma; D/V, 1.40 mm from the dura) using stereotactic coordinates at a rate of 30 nL/min.

For calcium imaging in vivo, we unilaterally injected 200 nL of AAV9-CaMKlla-Gcamp6f (9.37 × 10^13^ genome copies/mL, Vigene, China) into the mPFC of male C57BL/6 J mice (A/P, + 1.95 mm; M/L, − 0.40 mm from bregma; D/V, 1.40 mm from the dura).

After each injection, the needle was left in the brain for 5 min before being slowly withdrawn to prevent the virus from leaking out. Mice were rehoused in groups after recovery from the anesthetic, weighed daily until their weights had stabilized and given 10% glucose in the drinking water for 2–4 days postoperatively to aid recovery. Viral expression lasted for approximately 4 weeks (except for GCaMP6f expression), a period that maximized efficacy for our purposes. GCaMP6f expression lasted for approximately 3 weeks before GRIN lens implantation.

### GRIN lens implantation

Briefly, 3 weeks after GCaMP6f virus injection, mice were anesthetized with sodium pentobarbital/xylazine (sodium pentobarbital: 50 mg/kg; xylazine: 30 mg/kg, i.p.), maintained with 1–2% isoflurane and placed in a stereotaxic frame. Body temperature was continuously monitored and maintained at 37 °C. A 1.1-mm diameter hole was made in the skull (coordinates: A/P, + 1.95 mm, M/L, − 0.50 mm). Then, the brain tissue above the prelimbic cortex (D/V: 1.40 mm) was slowly aspirated using a 30-gauge needle with a blunt tip and sharpened tip wall. The needle was attached to a robotic surgical instrument, which included a three-axis motorized robotic arm. MATLAB-based software (https://github.com/liang-bo/AutoStereota) was used to set program parameters to remove the brain tissue above the imaging region. Then, a 1-mm diameter GRIN lens (ILW-100-P0460-055-NC, Go! Foton, USA) was directly implanted into the imaging region and secured to the skull using dental cement (Metabond S380, Parkell). After GRIN lens implantation, mice were housed singly.

### *Calcium imaging *in vivo

The specific construction of the miniScope was shown in a previous report [68]. The blue LED power ranged from 0.1 to 0.3 mW in the focal plane. The images (400 × 400 pixels) were acquired at a 10-Hz frame rate, and the pixel size was approximately 2.75 μm. Three to eight weeks after GRIN lens implantation, the mouse was anesthetized with sodium pentobarbital/xylazine (sodium pentobarbital: 50 mg/kg; xylazine: 30 mg/kg, i.p.) and mounted on the stereotaxic apparatus. The miniScope (including the base and main body), attached to the robotic surgical instrument, was mounted onto the mouse skull. The position of the miniScope above the GRIN lens was adjusted to obtain the best focus for the entire field of view. Then, dental cement was used to fix the miniScope base to the mouse head. After detaching the base with the main body of the miniScope, a protective cap was attached to the base with a locking screw. One week after base fixation, the mice were subjected to behavioral tests. Before behavioral tests, the main body of the miniScope was connected to the fixed base component through a locking screw. Prior to calcium imaging recording, the mouse was adapted to the miniScope for 10 min. Then, the mouse was subjected to behavior tasks for approximately 1 h, and the calcium imaging recording continued. After the behavioral task, the main body of the miniScope was detached from the base, and the mouse was returned to its home cage.

### Calcium imaging processing

All of the analysis procedures were performed in MATLAB 2021a (MathWorks, USA). The obtained raw data were transformed into a tiff stack, in which each slice was a frame of calcium signal. Then, a process of motion correction was performed to eliminate optical shifting based on an open-source script described in a previous report [69]. Extended constrained nonnegative matrix factorization (CNMF-E) [34] was applied to extract calcium signal traces (DF/F) and regions of interest (ROIs) of each recorded neuron. The calcium signal traces were low-pass filtered and deconvoluted by the integrated CNMF-E program. For the rising phase of calcium activity, a customized MATLAB script was used to mark the calcium events by the peak DF/F values. To track neurons on separated recording days, we applied the Cellreg [70] algorithm to match neurons from different days according to the distance and spatial correlations among them.

### Behavior annotation

The behavior tasks were recorded using a top-view camera (MER-132-30GM, DAHENG IMAGING). A data acquisition controller was used to set up the connection between the miniScope, host computer, and camera. The synchronization between calcium imaging and behavior recording was performed by the external transistor-transistor logic (TTL) signals from the data acquisition controller. The frame rate of the camera recording was the same as that of the miniScope recording. Mouse behavioral activities were analyzed manually with frame-by-frame annotation to define behavior time windows (behavior occurring: 1, behavior not occurring: 0). Distinct behavioral activities were annotated, such as poking, licking on the correct side that was rewarded, and licking on the incorrect side that was not rewarded. The criteria for these annotated behaviors are listed below: (1) Poking labeled the moment when the subject mouse poked the center port and the visual stimulus onset. (2) Correct side licking labeled the moment when the subject mouse chose the port at the side where the positive stimulus was presented and was rewarded by water (4–5 μl). (3) Incorrect side licking labeled the moment when the subject mouse chose the incorrect port followed by a timeout period (10 s). For the poking responses, time windows of − 0.5 to 1.5 s relative to visual stimulus onset at *t* = 0 s were defined, and for the reward-related responses, − 0.5 to 3.5 s relative to water delivery onset time windows were defined.

### Identification of behaviorally tuned neurons

For each testing stage, we identified behaviorally tuned neurons corresponding to each of the annotated behaviors (i.e., trigger, reward), following the same analytical steps as described below (also see Additional file 1: Fig. S2D, 2E): (1) For any given neuron n, we first calculated the similarity between calcium trace (DF/F) Cn and behavior vector B. Similarity was defined as the normalized inner product of two vectors, 2B∙Cn/(|B|^2^ +|Cn|^2^) [35, 36]. The value of similarity was bound between 0 and 1. A value of 1 indicated that these two vectors were identical, while a value of 0 indicated that they were completely different. (2) We then randomly shuffled the behavior epochs and calculated the similarity between the shuffled behavior vector and the calcium trace Cn for a given neuron. We repeated this permutation process 5000 times to generate a similarity distribution representing the similarity distribution histogram predicted by chance for a given neuron. (3) A neuron was defined as an “ON neuron” only if its actual similarity was greater than the 99.17% quantile of chance similarity distribution histogram generated by random shuffling. Consequently, ON neurons were those exhibiting significantly higher activities than those predicted by chance during the annotated behavior epochs. Conversely, a neuron was defined as an “OFF neuron” only if its actual similarity was lower than the 0.83% quantile of chance similarity distribution histogram generated by random shuffling. Therefore, OFF neurons exhibited significantly lower activity than that predicted by chance during the annotated behavior epochs. Neurons with similarity falling between the 0.83% and 99.17% quantiles of chance similarity distribution histogram generated by random shuffling were defined as “Other neurons.” For each mouse, we applied the calculations described above to all active neurons to identify the ON and OFF neurons correlated with each of the annotated behaviors during different testing stages. We defined each group of ON neurons (or OFF neurons) as one “neural ensemble”.

### Behavioral type decoding

We employed the widely used linear classifier with stochastic gradient descent (SGD) training in Python to test whether different behavioral types could be reliably predicted using calcium activity *z* scores of the identified neurons in each mouse at different stages of learning. For each mouse and each behavior type, we trained and tested a classifier using five types of calcium trace datasets: (1) calcium traces of all neurons; (2) calcium traces of the behavior-related ON and OFF neurons; (3) calcium traces of the behavior-related ON neurons and the randomly shuffled calcium traces of the behavior-related OFF neurons; (4) calcium traces of the behavior-related OFF neurons and the randomly shuffled calcium traces of the behavior-related ON neurons; and (5) randomly shuffled calcium traces of both behavior-related ON and OFF neurons. We applied a tenfold cross-validation procedure as in previous works [37, 71] to train and test the decoder. The original datasets (behavior vector and calcium traces of each neural group) were randomly divided into 10 equal-sized subsets. Nine of the ten subsamples were used for training the model, and the remaining subset was used for testing the decoder. The decoder predicts if the event occurred or not in each frame. This process was repeated ten times. The percentage of accurate classification incidence in the ten times was reported as the final balanced accuracy. The range of the balanced accuracy is from 0 to 1. The balanced accuracy = 1 represents perfect classification. Balanced accuracy = 0.5 represents the chance level.

### Quantitation of spatial distribution of neural ensembles

Moran’s Index is used to measure the spatial autocorrelation of all recorded different types of neurons. Global Moran’s I is a measure of the overall clustering of the spatial data. It is defined as

$$I=\frac NW\frac{\Sigma_{i=1}^N\Sigma_{j=1}^Nw_{ij}(x_i-\overline x)(x_j-\overline x)}{\Sigma_{j=1}^N{(x_i-\overline x)}^2}$$

*N* is the number of spatial units indexed by *i* and *j*; *x* of the analyzed ensemble neurons were set as 1, while the rest of neurons were set as 0; ‾*x* is the average *x* of all neurons; *w*_*ij*_ were set as inverse squared distance of each pair of neurons (*w*_*ii*_ = 0); *W* is the sum of all *w*_*ij*_.

Values of Moran’s I usually range from − 1 to + 1 (− 1 is perfect clustering of dissimilar groups; 0: perfect randomness; + 1: perfect clustering of similar groups. Only if the *p* value that corresponds to Moran’s I is less than 0.05, then we can reject the null hypothesis (H0: The data is randomly dispersed) and conclude that the data is spatially clustered together. All of Moran’s I analysis and transformed *p* values were performed in ArcGIS 10.7.

### Histology

Mice were anesthetized deeply and transcardially perfused with PBS, followed by 4% paraformaldehyde (PFA) dissolved in 0.1 M PB (pH 7.4). Brains were removed and postfixed overnight at 4 °C in 0.1 M PB containing 4% PFA and immersed in 10% followed by 30% sucrose in 0.01 M PBS (pH 7.4) for cryoprotection. Each brain was sectioned into serial slices (50-μm thick) through the mPFC region with a sliding microtome (CM1950, Leica, Germany). To present virus- or GRIN lens-targeted regions, coronal brain sections were washed in PBS three times (10 min each time). Sections were then exposed to 40,6-diamidino-2-phylindole, dihydrochloride (DAPI; Thermo Fisher Scientific, D1306) diluted 1:1000 in PBS containing 0.3% Triton X-100 at room temperature for 30 min. After washing in PBS three times (10 min each time), sections were mounted on slides with 80% glycerol solution and photographed. Anatomical regions were identified according to the Brain Atlas of Paxinos & Franklin (2001) and the Allen Institute Mouse Brain Atlas.

### Behavioral assays

The behavioral experiments were performed between 10:00 A.M. and 8:00 P.M. For acute chemogenetic inhibition experiments, saline or 5 mg/kg CNO dissolved in saline was delivered 30 min prior to each behavioral test.

### Visual water task

#### Visual discrimination task acquisition

The apparatus used in this study was the same as that used in a previous study [12], and the swimming paths were recorded with a digital camera mounted above the pool and analyzed offline with Ethovision XT 8.5 (Noldus, Info Tech, Wageningen, Netherlands). Two sessions consisting of 24 trials were executed per day. First, the mice must learn to find the hidden escape platform that is placed on the side of the positive stimulus (vertical sinusoidal grating, 0.12 cpd). A negative stimulus was a horizontal sinusoidal grating of the same SF. To exclude the potential effect of platform position, the side on which the platform was placed was pseudorandomly picked, and consecutive selection of one single side was limited to two times at most. If the mouse swam across the choice line toward the negative direction, the trial was considered incorrect. When the correct rate reached 100%, subsequent procedures were started. The training procedures for VPL consisted of three consecutive phases: pretraining VA assessment, VPL of VA, and posttraining VA reassessment.

#### VA assessments

For VA assessment, the procedure began with a low SF (0.12 cpd) grating. The SF was increased incrementally within blocks of trials in a step of 0.03 cpd when no less than 3 correct choices were made in 4 consecutive tests or when seven correct choices were made in a block of 10 consecutive trials. Otherwise, if the accuracy decreased below 70%, the SF was decreased. The threshold of SF was repeatedly assessed. Finally, an accuracy-SF curve was fitted, and the visual acuity of the mouse was determined as the grating SF corresponding to 70% accuracy.

#### VPL of VA

The training procedures were identical to the VA assessment described above. For VPL of VA, the grating SF was varied near the individual SF threshold (NSFT training) at 100% contrast within blocks of trials. Once a mouse failed to achieve 70% accuracy at the SF threshold, the SF was decreased in steps until an accuracy greater than 70% was achieved, and vice versa. Mice received NSFT training for 35 consecutive days, and two sessions were performed on a single day.

### Custom-designed training chamber

Mice were water restricted in their cages, and weight was maintained above 85% of the prerestriction body weight. The custom-designed training chamber used in this study and the steps of training to perform the task were similar to those in a previous study [72]. VPL training was conducted in a custom-designed chamber (36 × 17 × 29 cm, L × W × H), with three ports positioned along the front wall facing an LCD monitor. The chamber was separated into three connected areas by two dividers. The nose poke into each port was detected by the interruption of an infrared beam. Mice were trained to initiate a trial by poking the center port (trigger), and the positive stimulus (vertical sinusoidal grating, 0.12 cpd) and negative stimulus (horizontal sinusoidal grating, 0.12 cpd) were presented on two sides of the monitor. To exclude the potential effect of the reward port location, the side to which the positive stimulus was presented was pseudorandom, and consecutive presentation on one single side was limited to two times at most. Correct responses (licking the water spout at the side the positive stimulus) were rewarded by water (5 μl), whereas incorrect responses (licking at the incorrect side) were followed by a timeout period (10 s). Nonresponding within 20 s after stimulus presentation is referred to as an omission. The stimulus disappeared after the mice made the choice. After reaching a correct rate of ∼90% for three consecutive sessions (approximately 12 days), subsequent VPL procedures were started. The VPL procedures performed in the behavioral chamber were identical to those in the visual water task, except one session consisting of ~ 100 trials was executed per day, and mice received NSF training for 7 consecutive days.

## Quantification and statistical analysis

### Statistical analysis

All data are presented as the mean ± SEM. Statistical significance between two groups was analyzed by Student’s *t* test or one-way ANOVA with post hoc Tukey’s test. Differences were considered to be significant with a *P* value less than 0.05.

## Supplementary Information

**Additional file 1: Figure S1.** Related to Fig. 1 and Fig. 2. Histological map showing the area of the lesion in the mPFC and the effect of ablation on task acquisition. A. Reconstruction of the mPFC lesions. The largest and smallest lesion are shown in pale and dark shading respectively. B. The correction rate in the NMDA lesion and sham groups across training days when mice acquired the visual discrimination task in the water maze. NMDA, *n =* 11; sham, *n =* 5. C. The average learning curve for mice across training days in the training chamber. *n =* 12. **Figure S2.** Related to Fig. 3. Effect of miniScope carrying on mouse behaviors and identification of behaviorally tuned ON and OFF mPFC neurons. A. The correction rate for miniscope-carried and control mice. Miniscope: *n<*8; ctrl: *n=*7. B. The accuracy rate for miniscope-carried and control mice. Miniscope: *n=*8; ctrl: *n=*7. C. The reaction time for miniscope-carried and control mice. Miniscope: *n=*8; ctrl: *n=*7. D. Schematic diagram shows the process of calcium-behavior similarity comparison for a given neuron. E. Representative calcium-behavior similarity comparisons from three example On, OFF and Other neurons (red, blue, and gray, respectively). Histograms represent distributions of the calcium-behavior chance similarity, calculated from 5,000 shuffling of the behavior vector. Dashed vertical lines indicate the 0.83 (R_left_) and 99.17 (R_right_) percentiles of the chance similarity distribution. Solid vertical lines (R) represent the actually observed values for the calcium-behavior similarity. F. The averaged calcium activity of ON (red) and OFF (blue) neurons in poking (left) and reward (right)-related neural ensembles at the onset (red-dotted line) of each behavior (± 5 seconds). G. Histograms of calcium events aligned to poking behavior onset (red-dotted line) of the Pok-ON (left) and Rew-ON (right) groups. H. Left: schematic of interbehavior overlap between Pok-OFF and Rew-OFF neural ensembles. Right: calcium events per trial aligned to poking behavior onset (black arrow) are shown from an example Pok-OFF neuron (top), an example Pok-OFF and Rew-OFF neuron (middle) and an example Rew-OFF neuron (bottom). The blue arrow shows when the reward behavior onset. Data are represented as the mean ± SEM. ∗, *p <* 0.05; ∗∗, *p <* 0.01; ∗∗∗, *p <* 0.005; ∗∗∗∗, *p <* 0.0001; n.s., not significant (*p* > 0.05). **Figure S3.** Related to Fig. 4. Neurons only showed an “ON” response after visual stimuli were presented. A. The averaged calcium activity of correct (left) and incorrect (right) trials at the onset (red-dotted line) of poking behavior (± 5 seconds) of Poking-ON neurons only showed an “ON” response after visual stimuli were presented. B. Histograms of calcium events aligned to poking behavior onset (red-dotted line) in correct (left) and incorrect (right) trials of Poking-ON neurons only showed an “ON” response after visual stimuli are presented. **Figure S4.** Location preference of Rewarded neurons. A. Proportions of neurons with different location preference at Low SF stage (left) and the NSF stage (right). B. Top: The mean z score during the reward time window at left or right ports of the example “Rewarded” neurons. Bottom: Raster plot of the averaged z score of individual “Rewarded” neurons at left or right ports. Data are represented as the mean ± SEM. ∗∗, *p <* 0.01. **Figure S5.** Related to Fig. 6. mPFC pyramidal neurons show similar heterogeneous responses to predictive cues during the NSF training stage. A Raster plot of the averaged z score of individual Poking-ON and Poking-OFF neurons at the onset of poking behavior (5 s before and 5 s after), sorted by the time of maximal activities (ON neurons) or minimal activities (OFF neurons). B. Spatial distributions of Pok-ON and Pok-OFF neurons from the same representative mouse; scale bar, 100 μm. C. Raster plot of the averaged z score of individual Reward-ON and Reward-OFF neurons at the onset of reward behavior (5 s before and 5 s after), sorted by the time of maximal activities (ON neurons) or minimal activities (OFF neurons). D. Spatial distributions of Rew-ON and Rew-OFF neurons from the same representative mouse; scale bar, 100 μm. E. Left: schematic of interbehavior overlap between Pok-ON and Rew-ON neural ensembles. Right: calcium events per trial aligned to poking behavior onset (black arrow) are shown from an example Pok-ON neuron (top) and an example Rew-ON neuron (bottom). The blue arrow shows when the reward behavior onset. F. Left: schematic of interbehavior overlap between Pok-OFF and Rew-OFF neural ensembles. Right: calcium events per trial aligned to poking behavior onset (black arrow) are shown from an example Pok-OFF neuron (top) and an example Rew-OFF neuron (bottom). The blue arrow shows when the reward behavior onset. **Figure S6.** Related to Fig. 6. Four neuronal ensembles could also be identified during the NSF training stage. A. Histograms of calcium events aligned to poking behavior onset (red line) of “Trigger-like” (top) and “Attention-like” (bottom) groups in correct (left) and incorrect (right) trials. B. The mean z score during the poking time window on correct, incorrect and omission trials of the “Trigger-like” (left) and “Attention-like” (right) groups. Tri-like: *n=*27; Att-like: *n=*20. C. Calcium events per correct (left) and incorrect (right) trial aligned to poking behavior onset (arrow) are shown from an example “Trigger-like” neuron (top) and an example “Attention-like” neuron (bottom). D. Histograms of calcium events aligned to licking behavior onset (red line) of “Reward-pursuing” (top) and “Rewarded” (bottom) groups in correct (left) and incorrect (right) trials. E. The mean z score during the poking time window on correct, incorrect and omission trials of “Reward-pursuing” (left) and “Rewarded” (right) groups. Re-pu: *n=*46; Rewarded: *n=*81. F. Calcium events per correct (left) and incorrect (right) trial aligned to licking behavior onset (arrow) are shown from an example “Reward-pursuing” neuron (top) and two example “Rewarded” neurons (middle and bottom). Data are represented as the mean ± SEM. ∗, *p <* 0.05; ∗∗, *p <* 0.01; ∗∗∗, *p <* 0.005; ∗∗∗∗, *p <* 0.0001; n.s., not significant (*p* > 0.05). **Figure S7.** The decoding accuracy after balancing the neuron number in ON and OFF groups. A. Decoding analyses for poking behavior using calcium activity from balanced ON + shuffled OFF neurons and shuffled ON + OFF neurons. *n=*5. B. Decoding analyses for reward behavior using calcium activity from balanced ON + shuffled OFF neurons and shuffled ON + OFF neurons. *n=*5. Data are represented as the mean ± SEM. ∗∗, *p <* 0.01. **Figure S8.** The ROIs of neurons at the two different stages in an example mouse. Top: the regions of interest (ROIs) at the two different stages in an example mouse; scale bar: 100 μm. Bottom: enlarged view of area indicated by the yellow box in the top figures; scale bar: 50 μm. Red arrow, neurons only activated at low SF stage. Green arrow, neurons only activated at NSF stage. **Figure S9.** Related to Fig. 7. The neuronal proportion on the first NSF training day and the transformation of neural ensembles in or between the two different stages. A. Proportions of each ensemble at low SF training stage (left), the first NSF training day (middle) and the fifth NSF training day (right). B. Specific transformation number of the tracked neurons between two different stages. C. The transformed proportion of neurons between two halves of the same stage (left: Low SF stage; right: NSF training stage).

## Data Availability

All data generated or analyzed during this study are included in this published. article, its supplementary information files, and publicly available repositories. (https://osf.io/9sbpq/files/osfstorage).
