# Supplementary material for: Neural ensembles in the murine medial prefrontal cortex process distinct information during visual perceptual learning
Source: BMC Biol. 2023 Feb 24;21:44. doi: 10.1186/s12915-023-01529-x (PMC9960446; doi:10.1186/s12915-023-01529-x)
Supplement: Supplementary file 1 — Additional file 1: Figure S1. Related to Fig. 1 and Fig. 2. Histological map showing the area of the lesion in the mPFC and the effect of ablation on task acquisition. A. Reconstruction of the mPFC lesions. The largest and smallest lesion are shown in pale and dark shading respectively. B. The correction rate in the NMDA lesion and sham groups across training days when mice acquired the visual discrimination task in the water maze. NMDA, n = 11; sham, n = 5. C. The average learning curve for mice across training days in the training chamber. n = 12. Figure S2. Related to Fig. 3. Effect of miniScope carrying on mouse behaviors and identification of behaviorally tuned ON and OFF mPFC neurons. A. The correction rate for miniscope-carried and control mice. Miniscope: n<8; ctrl: n=7. B. The accuracy rate for miniscope-carried and control mice. Miniscope: n=8; ctrl: n=7. C. The reaction time for miniscope-carried and control mice. Miniscope: n=8; ctrl: n=7. D. Schematic diagram shows the process of calcium-behavior similarity comparison for a given neuron. E. Representative calcium-behavior similarity comparisons from three example On, OFF and Other neurons (red, blue, and gray, respectively). Histograms represent distributions of the calcium-behavior chance similarity, calculated from 5,000 shuffling of the behavior vector. Dashed vertical lines indicate the 0.83 (Rleft) and 99.17 (Rright) percentiles of the chance similarity distribution. Solid vertical lines (R) represent the actually observed values for the calcium-behavior similarity. F. The averaged calcium activity of ON (red) and OFF (blue) neurons in poking (left) and reward (right)-related neural ensembles at the onset (red-dotted line) of each behavior (± 5 seconds). G. Histograms of calcium events aligned to poking behavior onset (red-dotted line) of the Pok-ON (left) and Rew-ON (right) groups. H. Left: schematic of interbehavior overlap between Pok-OFF and Rew-OFF neural ensembles. Right: calcium events per t [file 12915_2023_1529_MOESM1_ESM.docx]

**Supplementary information**

**Neural ensembles in the murine medial prefrontal cortex process distinct information during visual perceptual learning**

**Zhenni Wang ^1,3^, Shihao Lou^1,3^, Xiao Ma^1^, Hui Guo^1^, Yan Liu^1^, Wenjing Chen^1^, Dating Lin^2^ and Yupeng Yang^1,4*^**

**Zhenni Wang ^1,3^, Shihao Lou^1,3^, Xiao Ma^1^, Hui Guo^1^, Yan Liu^1^, Wenjing Chen^1^, Dating Lin^2^ and Yupeng Yang^1,4*^**

^1^Division of Life Sciences and Medicine, University of Science and Technology of China, Hefei, 230027, China

^2^Intramural Research Program, National Institute on Drug Abuse, National Institutes of Health, Baltimore, MD 21224, USA

^3^These authors contributed equally

^4^Lead contact

* Correspondence: yangyp@ustc.edu.cn


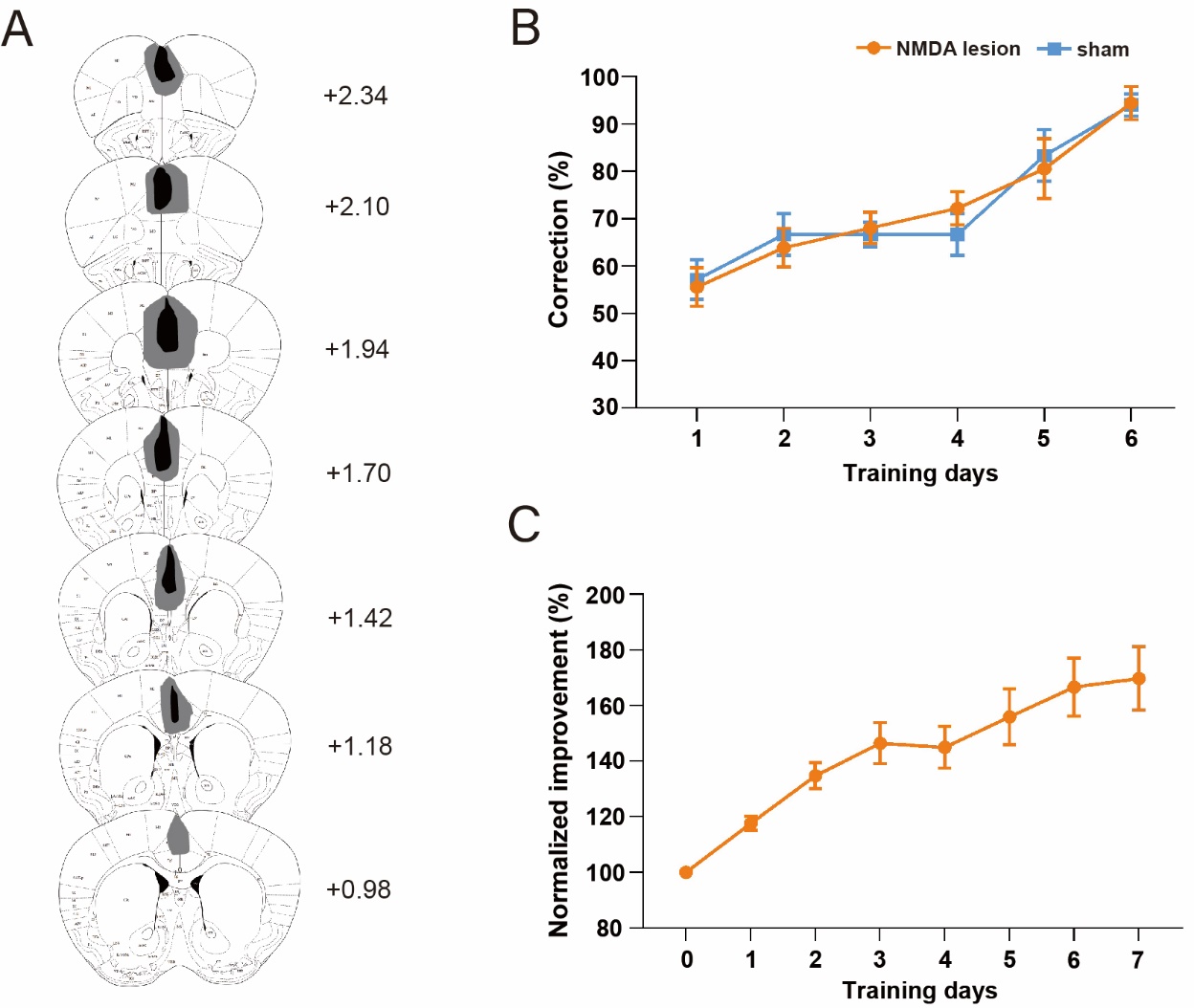


Figure S1. Related to Figure 1 and Figure 2. Histological map showing the area of the lesion in the mPFC and the effect of ablation on task acquisition.

1. Reconstruction of the mPFC lesions. The largest and smallest lesion are shown in pale and dark shading

respectively.

B. The correction rate in the NMDA lesion and sham groups across training days when mice acquired the visual discrimination task in the water maze. NMDA, n = 11; sham, n = 5.

C. The average learning curve for mice across training days in the training chamber. n = 12.


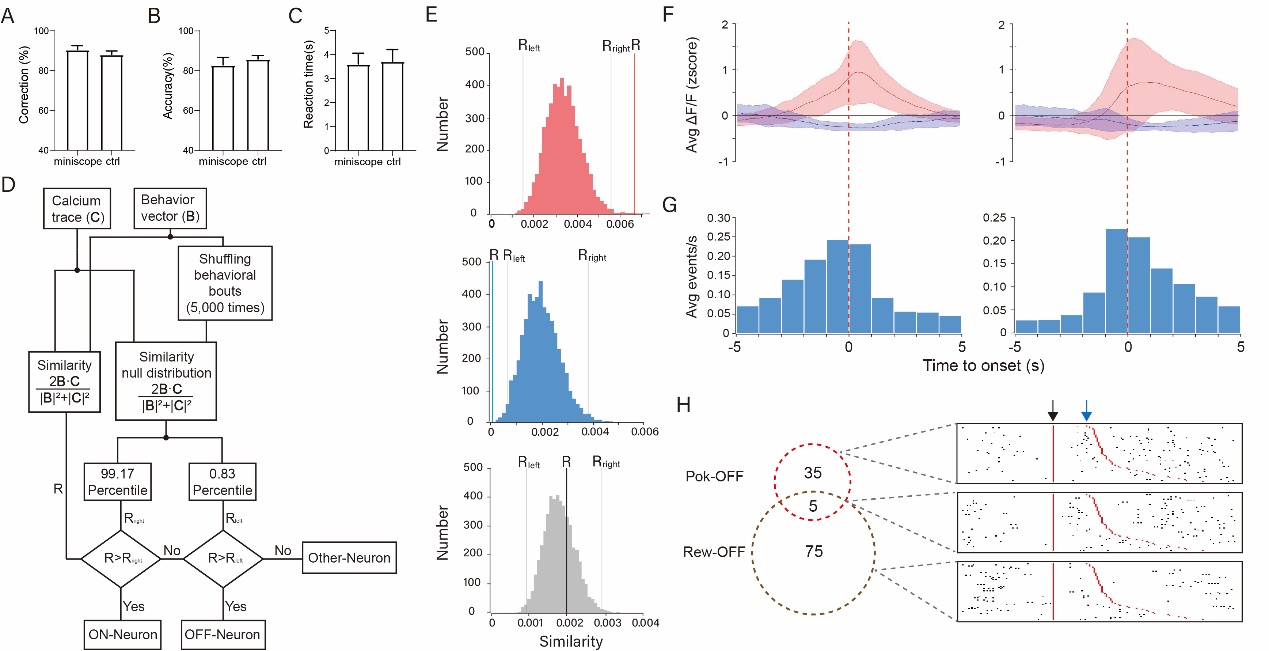
Figure S2. Related to Figure 3. Effect of miniScope carrying on mouse behaviors and identification of behaviorally tuned ON and OFF mPFC neurons.

A. The correction rate for miniscope-carried and control mice. Miniscope: n=8; ctrl: n=7.

B. The accuracy rate for miniscope-carried and control mice. Miniscope: n=8; ctrl: n=7.

C. The reaction time for miniscope-carried and control mice. Miniscope: n=8; ctrl: n=7.

D. Schematic diagram shows the process of calcium-behavior similarity comparison for a given neuron.

E. Representative calcium-behavior similarity comparisons from three example On, OFF and Other neurons (red, blue, and grey, respectively). Histograms represent distributions of the calcium-behavior chance similarity, calculated from 5,000 shuffling of the behavior vector. Dashed vertical lines indicate the 0.83 (R_left_) and 99.17 (R_right_) percentiles of the chance similarity distribution. Solid vertical lines (R) represent the actually observed values for the calcium-behavior similarity.

F. The averaged calcium activity of ON (red) and OFF (blue) neurons in poking (left) and reward (right)-related neural ensembles at the onset (red-dotted line) of each behavior (± 5 seconds).

G. Histograms of calcium events aligned to poking behavior onset (red-dotted line) of the Pok-ON (left) and Rew-ON (right) groups.

H. Left: schematic of interbehavior overlap between Pok-OFF and Rew-OFF neural ensembles. Right: calcium events per trial aligned to poking behavior onset (black arrow) are shown from an example Pok-OFF neuron (top), an example Pok-OFF and Rew-OFF neuron (middle) and an example Rew-OFF neuron (bottom). The blue arrow shows when the reward behavior onset.

Data are represented as the mean ± SEM. ∗, p < 0.05; ∗∗, p < 0.01; ∗∗∗, p < 0.005; ∗∗∗∗, p < 0.0001; n.s., not significant (p > 0.05).


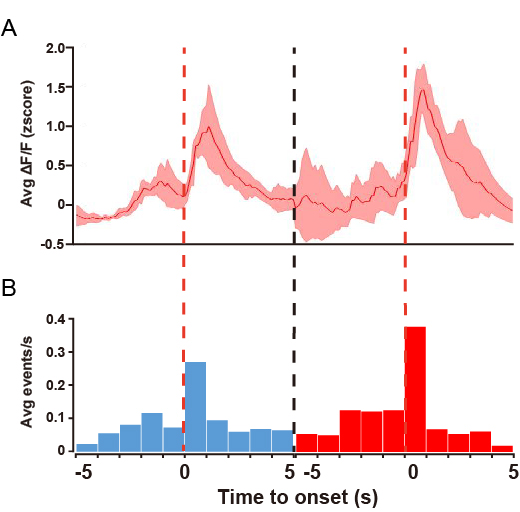


Figure S3. Related to Figure 4. Neurons only showed an “ON” response after visual stimuli were presented.

A. The averaged calcium activity of correct (left) and incorrect (right) trials at the onset (red-dotted line) of poking behavior (± 5 seconds) of Poking-ON neurons only showed an “ON” response after visual stimuli were presented.

B. Histograms of calcium events aligned to poking behavior onset (red-dotted line) in correct (left) and incorrect (right) trials of Poking-ON neurons only showed an “ON” response after visual stimuli are presented.


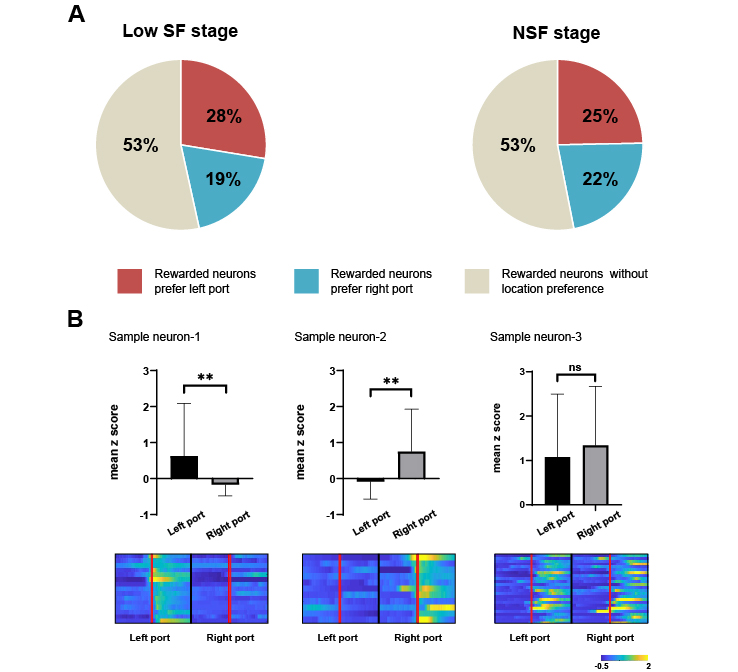


Figure S4. Location preference of Rewarded neurons.

1. Proportions of neurons with different location preference at Low SF stage (left) and the NSF stage (right).

B. Top: The mean z score during the reward time window at left or right ports of the example “Rewarded” neurons. Bottom: Raster plot of the averaged z score of individual “Rewarded” neurons at left or right ports.

Data are represented as the mean ± SEM. ∗∗, p < 0.01.


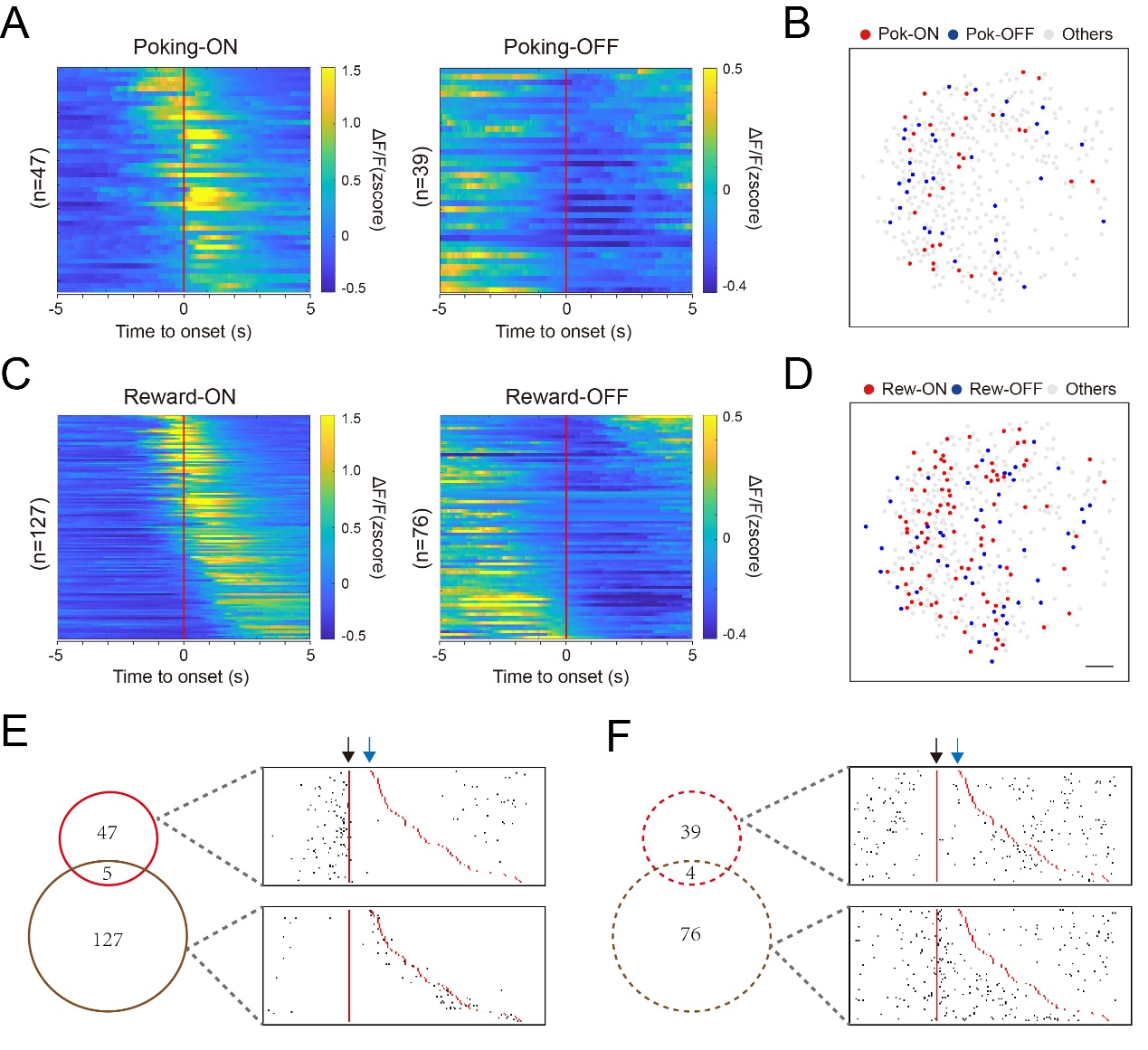
Figure S5. Related to Figure 6. mPFC pyramidal neurons show similar heterogeneous responses to predictive cues during the NSF training stage.

A Raster plot of the averaged z score of individual Poking-ON and Poking-OFF neurons at the onset of poking behavior (5 s before and 5 s after), sorted by the time of maximal activities (ON neurons) or minimal activities (OFF neurons).

B. Spatial distributions of Pok-ON and Pok-OFF neurons from the same representative mouse; scale bar, 100 μm.

C. Raster plot of the averaged z score of individual Reward-ON and Reward-OFF neurons at the onset of reward behavior (5 s before and 5 s after), sorted by the time of maximal activities (ON neurons) or minimal activities (OFF neurons).

D. Spatial distributions of Rew-ON and Rew-OFF neurons from the same representative mouse; scale bar, 100 μm.

E. Left: schematic of interbehavior overlap between Pok-ON and Rew-ON neural ensembles. Right: calcium events per trial aligned to poking behavior onset (black arrow) are shown from an example Pok-ON neuron (top) and an example Rew-ON neuron (bottom). The blue arrow shows when the reward behavior onset.

F. Left: schematic of interbehavior overlap between Pok-OFF and Rew-OFF neural ensembles. Right: calcium events per trial aligned to poking behavior onset (black arrow) are shown from an example Pok-OFF neuron (top) and an example Rew-OFF neuron (bottom). The blue arrow shows when the reward behavior onset.


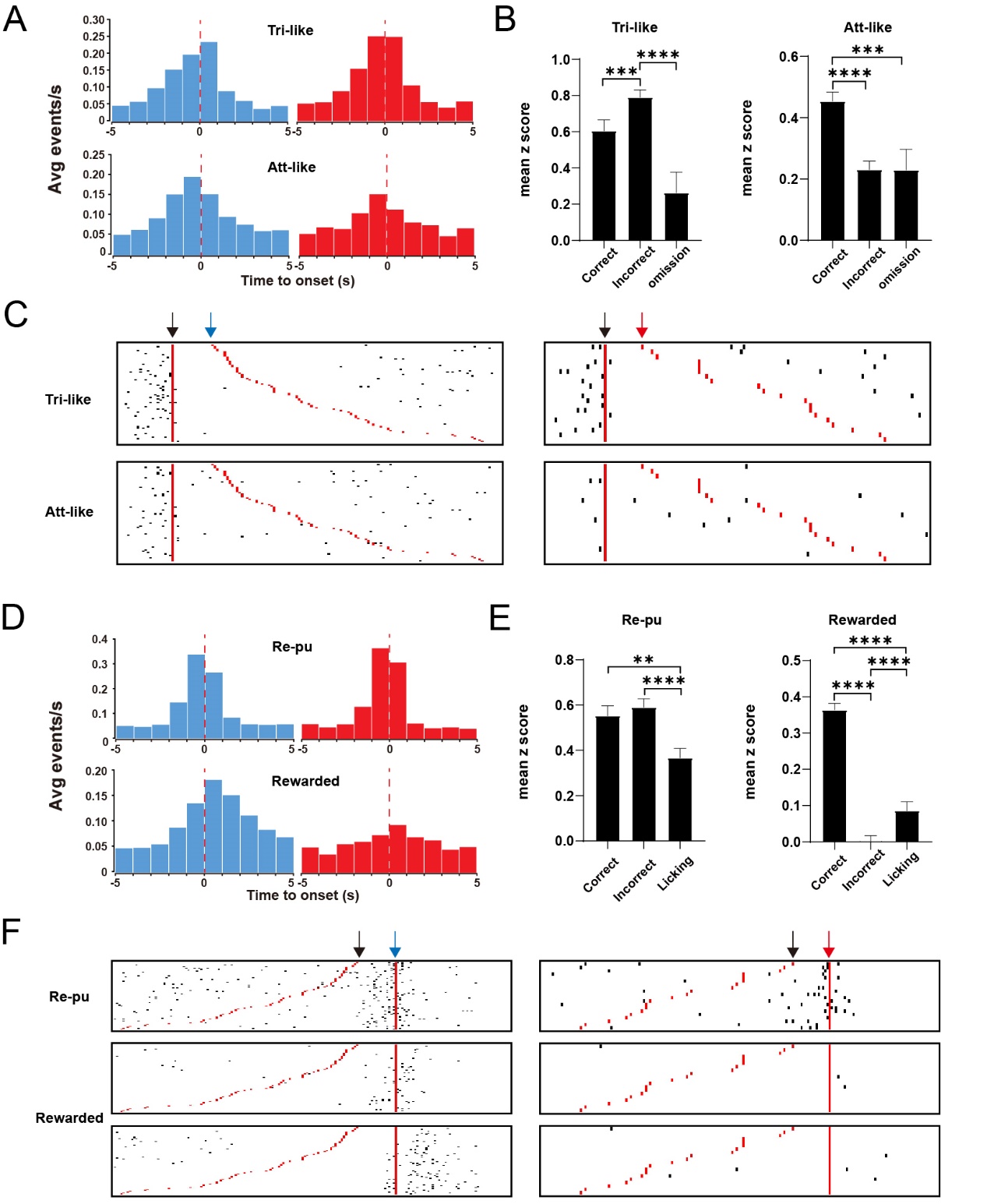


Figure S6. Related to Figure 6. Four neuronal ensembles could also be identified during the NSF training stage.

A. Histograms of calcium events aligned to poking behavior onset (red line) of “Trigger-like” (top) and “Attention-like” (bottom) groups in correct (left) and incorrect (right) trials.

B. The mean z score during the poking time window on correct, incorrect and omission trials of the “Trigger-like” (left) and “Attention-like” (right) groups. Tri-like: n=27; Att-like: n=20.

C. Calcium events per correct (left) and incorrect (right) trial aligned to poking behavior onset (arrow) are shown from an example “Trigger-like” neuron (top) and an example “Attention-like” neuron (bottom).

D. Histograms of calcium events aligned to licking behavior onset (red line) of “Reward-pursuing” (top) and “Rewarded” (bottom) groups in correct (left) and incorrect (right) trials.

E. The mean z score during the poking time window on correct, incorrect and omission trials of “Reward-pursuing” (left) and “Rewarded” (right) groups. Re-pu: n=46; Rewarded: n=81.

F. Calcium events per correct (left) and incorrect (right) trial aligned to licking behavior onset (arrow) are shown from an example “Reward-pursuing” neuron (top) and two example “Rewarded” neurons (middle and bottom).

Data are represented as the mean ± SEM. ∗, p < 0.05; ∗∗, p < 0.01; ∗∗∗, p < 0.005; ∗∗∗∗, p < 0.0001; n.s., not significant (p > 0.05).


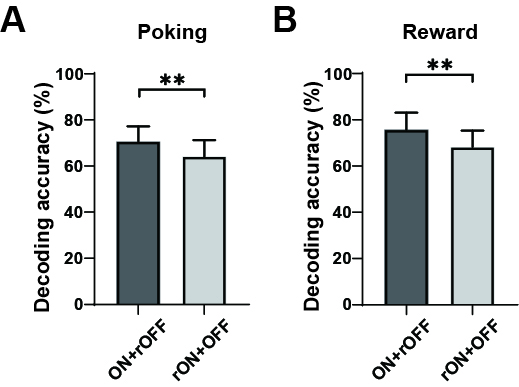


Figure S7. The decoding accuracy after balancing the neuron number in ON and OFF groups.

A. Decoding analyses for poking behavior using calcium activity from balanced ON + shuffled OFF neurons and shuffled ON + OFF neurons. n=5.

B. Decoding analyses for reward behavior using calcium activity from balanced ON + shuffled OFF neurons and shuffled ON + OFF neurons. n=5.

Data are represented as the mean ± SEM. ∗∗, p < 0.01.


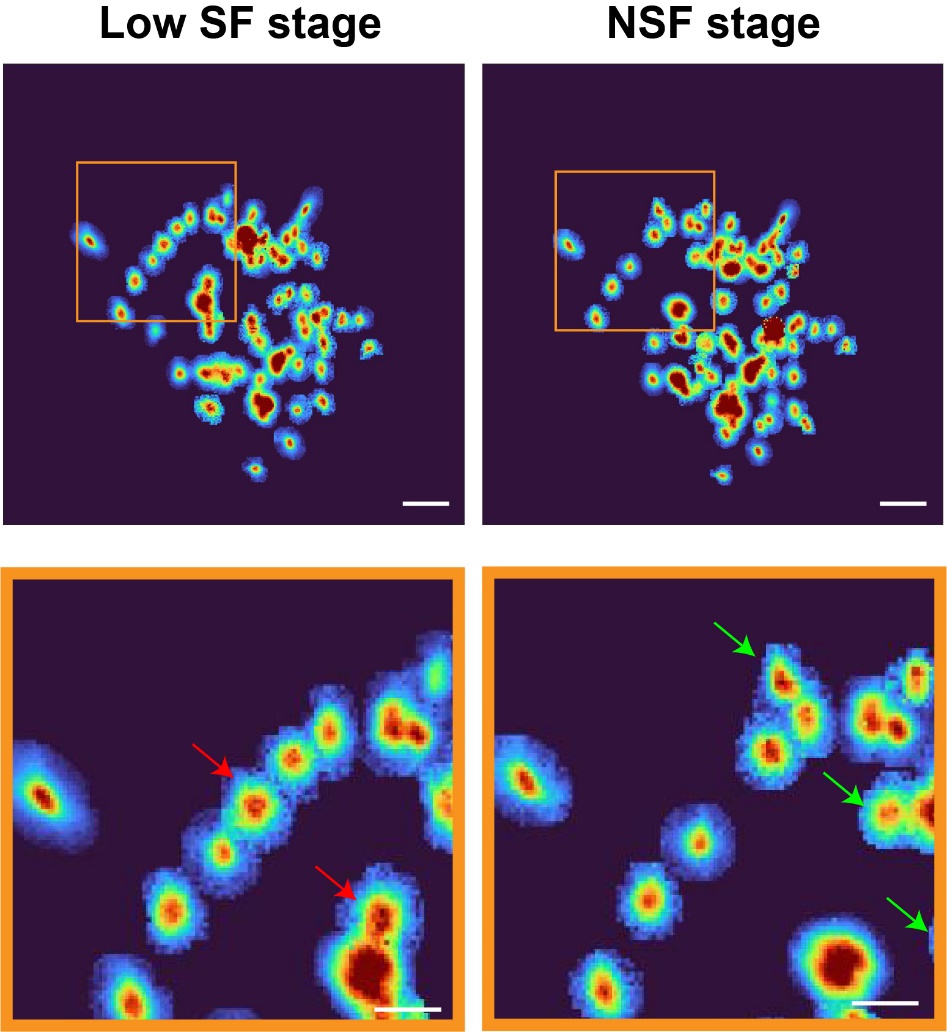


Figure S8. The ROIs of neurons at the two different stages in an example mouse.

Top: the regions of interest (ROIs) at the two different stages in an example mouse; scale bar: 100 μm. Bottom: enlarged view of area indicated by the yellow box in the top figures; scale bar: 50 μm. Red arrow, neurons only activated at low SF stage. Green arrow, neurons only activated at NSF stage.


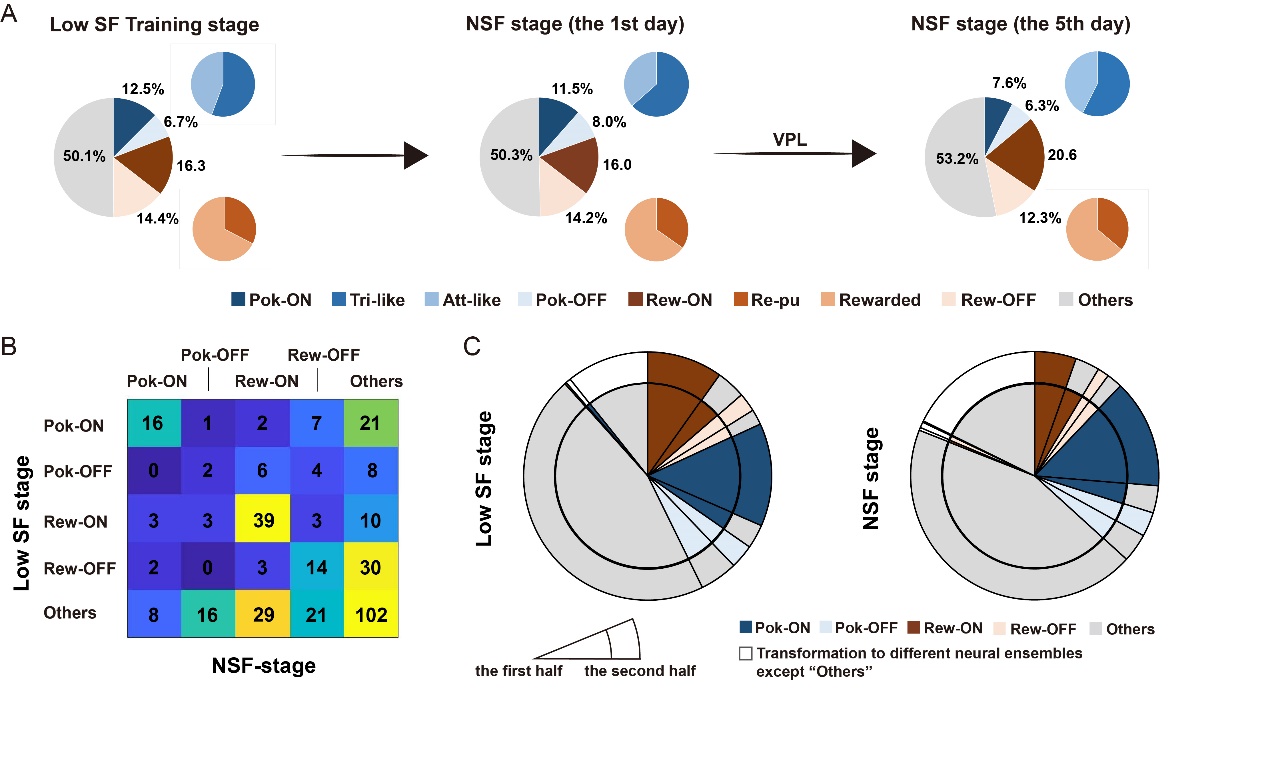


Figure S9. Related to Figure 7. The neuronal proportion on the first NSF training day and the transformation of neural ensembles in or between the two different stages.

A. Proportions of each ensemble at low SF training stage (left), the first NSF training day (middle) and the fifth NSF training day (right).

B. Specific transformation number of the tracked neurons between two different stages.

C. The transformed proportion of neurons between two halves of the same stage (left: Low SF stage; right: NSF training stage).
